# Prevention of Retinal Degeneration in a Rat Model of Smith-Lemli-Opitz Syndrome

**DOI:** 10.1038/s41598-018-19592-8

**Published:** 2018-01-19

**Authors:** Steven J. Fliesler, Neal S. Peachey, Josi Herron, Kelly M. Hines, Nadav I. Weinstock, Sriganesh Ramachandra Rao, Libin Xu

**Affiliations:** 1 Research Service, VA Western New York Healthcare System, Buffalo, NY USA; 2 Departments of Ophthalmology and Biochemistry, and Neuroscience Program, Jacobs School of Medicine & Biomedical Sciences, University at Buffalo- The State University of New York (SUNY), Buffalo, NY USA; 3 SUNY Eye Institute, Buffalo, NY USA; 4 Research Service, Louis Stokes Cleveland VA Medical Center, Cleveland, OH USA; 5 Department of Ophthalmic Research, Cole Eye Institute, Cleveland Clinic Foundation, Cleveland, OH USA; 6 Department of Ophthalmology, Cleveland Clinic Lerner College of Medicine of Case Western Reserve University, Cleveland, OH USA; 7 Department of Medicinal Chemistry, School of Pharmacy, University of Washington, Seattle, WA USA; 8 Hunter James Kelly Research Institute, Jacobs School of Medicine & Biomedical Sciences, University at Buffalo- The State University of New York (SUNY), Buffalo, NY USA

## Abstract

Smith-Lemli-Opitz Syndrome (SLOS) is a recessive human disease caused by defective cholesterol (CHOL) synthesis at the level of DHCR7 (7-dehydrocholesterol reductase), which normally catalyzes the conversion of 7-dehydrocholesterol (7DHC) to CHOL. Formation and abnormal accumulation of 7DHC and 7DHC-derived oxysterols occur in SLOS patients and in rats treated with the DHCR7 inhibitor AY9944. The rat SLOS model exhibits progressive and irreversible retinal dysfunction and degeneration, which is only partially ameliorated by dietary CHOL supplementation. We hypothesized that 7DHC-derived oxysterols are causally involved in this retinal degeneration, and that blocking or reducing their formation should minimize the phenotype. Here, using the SLOS rat model, we demonstrate that combined dietary supplementation with CHOL plus antioxidants (vitamins E and C, plus sodium selenite) provides better outcomes than dietary CHOL supplementation alone with regard to preservation of retinal structure and function and lowering 7DHC-derived oxysterol formation. These proof-of-principle findings provide a translational, pre-clinical framework for designing clinical trials using CHOL-antioxidant combination therapy as an improved therapeutic intervention over the current standard of care for the treatment of SLOS.

## Introduction

Smith-Lemli-Opitz syndrome (SLOS) represents one of several known complex recessive human disorders caused by an inborn error in cholesterol (CHOL) biosynthesis^1,2^. The specific genetic defect involves mutations in the gene that encodes 7-dehydrocholesterol reductase (DHCR7; also known as 3β-hydroxysterol Δ^7^-reductase; EC 1.3.1.21; OMIM #602858), the enzyme that catalyzes the conversion of 7-dehydrocholesterol (7DHC), the immediate biogenic precursor of CHOL, to CHOL^3,4^. This results in the abnormal and excessive accumulation of 7DHC (and its isomer, 8DHC), and concomitant reduction in the levels of CHOL, in the tissues and body fluids of affected individuals^5^. Hence, 7DHC accumulation is a prominent biomarker for SLOS. This hereditary disease is characterized by a constellation of phenotypic abnormalities (dysmorphologies) as well as functional deficits, some of which have been ascribed to CHOL deficiency during critical periods of embryonic development, or factors other than CHOL deficiency *per se*^1,2,6^. Prior studies^7^ have shown that 7DHC is the mostly readily oxidizable lipid known, resulting in the formation of more than a dozen structurally distinct and unique oxysterols (formed exclusively from 7DHC, but not other sterols)^8,9^, some of which are extremely toxic to cells, while others are more benign^10,11^. Furthermore, many 7DHC-derived oxysterols or oxysterol precursors are highly electrophilic, such as 7DHC-5α,6α-epoxide and 5α,6α-epoxycholest-7-en-3β,9α-diol (Compound 1 in^8^), which could damage proteins by forming adducts with their nucleophilic residues (see *Discussion*). It has been demonstrated that 7DHC itself behaves in a manner quite similar to that of CHOL, with respect to its ability to pack into lipid bilayers^12,13^ and to form “lipid rafts”^14,15^. Hence, it has been proposed that 7DHC-derived oxysterols, rather than 7DHC *per se*, may be causative in the pathobiology of this CHOL biosynthesis disorder^16–18^.

An animal model of SLOS has been created by treating albino Sprague-Dawley rats with AY9944, a relatively selective inhibitor of DHCR7, starting prenatally and continuing over the course of several weeks of postnatal life^19–21^. This “AY9944 rat model” of SLOS exhibits the key biochemical hallmarks as well as some of the phenotypic characteristics of SLOS. Over the course of the first 2–3 postnatal months, such rats undergo a progressive and irreversible retinal degeneration, characterized by photoreceptor dysfunction, death and drop-out, and defective macrophagy (autophagy and/or heterophagy) in the retinal pigment epithelium (RPE)^16,18,20^. Correlated with these pathological aspects of retinal cell biology are a host of parallel changes (or sequelae) beyond the initial CHOL biosynthetic defect, *e*.*g*., oxidation of lipids and proteins, marked glycerophospholipid and fatty acid profile changes, and marked dysregulation of gene expression, including up-regulation of genes and signaling pathways involved in cell death and oxidative stress and down-regulation of genes that are cytoprotective^16,18,22–25^. Hence, while the initial defect in SLOS appears to be simple and monogenic, the underlying pathobiology is multi-faceted and complex. Consistent with the *in vivo* observation that retinal cell death in the AY9944 rat model of SLOS is almost exclusively restricted to photoreceptor cells (rods and cones), rather than other retinal neuronal or glial cell types^25^, more recent *in vitro* studies, using cultured retina-derived immortalized cell lines, have indicated that photoreceptors are inherently far more susceptible to the cytotoxic effects of 7DHC-derived oxysterols than are RPE or Müller glial cells^11^. Importantly, studies from our labs have demonstrated the formation and persistent steady-state levels of 7DHC-derived oxysterols in the retina as well as other tissues (liver, brain, blood) in the AY9944 rat model of SLOS, whereas such oxysterols are virtually absent in the tissues of age-matched control rats^9,24^. Indeed, such oxysterols have been detected in appreciable levels in other animal and cellular models of SLOS^17,26–29^ as well as in plasma from human SLOS patients^30,31^.

The standard of care for treating SLOS patients is CHOL supplementation therapy^32,33^. However, the efficacy of this mode of therapeutic intervention is extremely variable and, in many cases, poor (see *Discussion*). Previously, using the AY9944 rat model of SLOS, we showed that dietary CHOL supplementation (*i*.*e*., feeding a rodent chow containing 2%, by wt., CHOL) resulted in a marked preservation of retinal electrophysiological function, particularly for the cone-driven pathway, and a near normalization of the CHOL levels (as well as significant decrease in 7DHC levels) in the retina, but did not significantly abate the histological degeneration^21^. Notably, these studies were performed well before the reports regarding the marked susceptibility of 7DHC to oxidation and the structures and cytotoxicity of 7DHC-derived oxysterols were elucidated. We hypothesized that if such oxysterols are key causative agents in the observed retinal degeneration in the AY9944 rat model of SLOS, as well as in the pathogenesis of SLOS in humans, then augmenting the CHOL supplementation approach with suitable antioxidants should block or diminish the formation of such oxysterols and thereby minimize the severity of the pathology^16,18,34,35^. In the present study, we tested that hypothesis, basing our antioxidant formulation on studies by Chen and Tappel^36^, which showed marked efficacy of high dietary levels of vitamins E (tocopherols) and C (ascorbic acid) plus selenium (Se) in protecting rat tissues and their constituent proteins from oxidative damage *in vivo*. The results obtained are consistent with our original hypothesis, and have important translational implications for improving the clinical management of SLOS patients.

## Results

### Combined CHOL-antioxidant dietary supplementation protects the retina from histological degeneration

**Figure 1:**
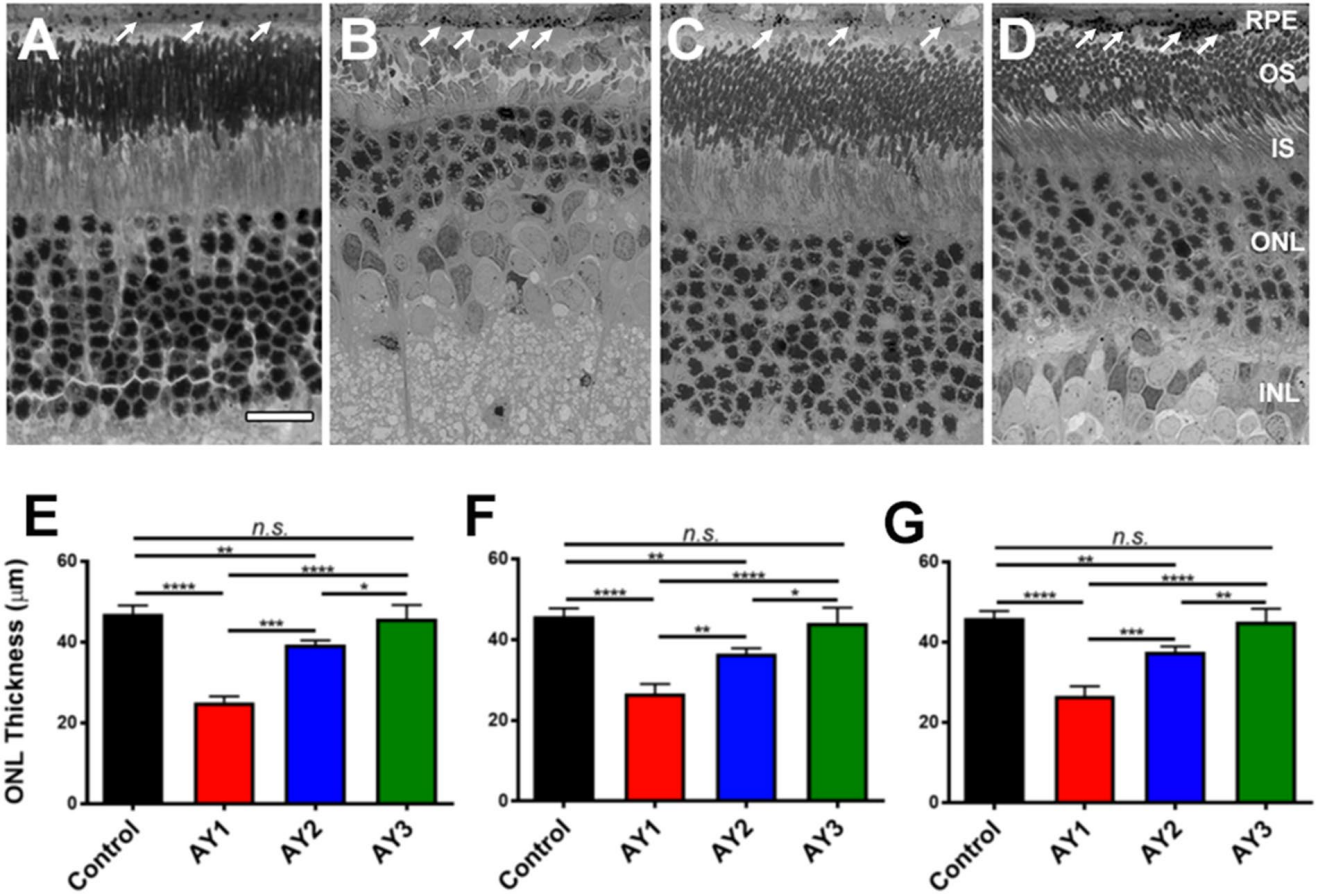
Retinal histology (*upper panels*, *A*–*D*) and quantitative morphometric analysis of ONL thickness (*lower panels*, *E*–*G*) of control *vs*. AY9944-treated rats on various diets. Light microscopy images (resin embedment, Toluidine blue stain; 40X objective) at age PN 80 days, under the following conditions: (**A**) Untreated rat fed a standard rodent diet (C1 group; *black*); (**B**) AY9944-treated rat fed a CHOL-free rodent diet (AY1 group; *red*); (**C**) AY9944-treated rat fed high-CHOL diet (AY2 group; *blue*); and (**D**) AY9944-treated rat fed high-CHOL diet supplemented with antioxidants (AY3 group; *green*). Presumed phagosomes in RPE are denoted by white arrows. *Abbreviations*: RPE, retinal pigment epithelium; OS, outer segment layer; IS, inner segment layer; ONL, outer nuclear layer; INL, inner nuclear layer. Scale bar (*panel* A, for all panels), 20 μm. ONL thickness measurements from (**E**) superior hemisphere, (**F**) inferior hemisphere, and (**G**) combined mean values (both hemispheres, ±S.E.M.; n = 3–4 biological replicates, n = 10 technical replicates, each condition), along the vertical meridian. Statistical significance (one-way ANOVA): *p < 0.05, **p < 0.01, ***p < 0.005, ****p < 0.001; n.s., not significant.

Figure 1 (*upper* panels, A–D) shows representative histological images illustrating the morphological appearance of the outer retina (superior equatorial zone, along the vertical meridian) in rats at postnatal 80 days (PN 80), comparing untreated control rats fed a standard rodent chow (which is CHOL-free) *vs*. AY9944-treated rats maintained on the three dietary regimens employed in this study: **AY1**, standard CHOL-free diet; **AY2**, high-CHOL diet; and **AY3**, high-CHOL diet supplemented with the antioxidants vitamin E, vitamin C, and sodium selenite (see *Methods* for detail). Note the healthy appearance and tight packing of the photoreceptor cells, the alignment of the rod outer segments (OS), and the normal appearance of the outer nuclear layer (ONL) with its 11–12 vertical rows of photoreceptor nuclei in the image of the retina from an untreated control rat (Fig. 1A). The RPE also contains a relatively modest number of phagosomes— ingested tips of OS shed by the adjacent photoreceptors— consistent with the fact that eyes were harvested in the late morning, at least 4 h after light onset. By stark contrast, the micrograph of a retina from an AY9944-treated rat fed the same standard rodent chow (Fig. 1B) exhibits marked photoreceptor degeneration, with truncated and poorly aligned rod inner segments (IS) and OS, and an ONL that has only 3–4 vertical rows of photoreceptor nuclei, indicative of massive death and dropout of photoreceptor cells. The remnant photoreceptors have histological features (shape, size, distribution, and nuclear position and appearance) consistent with being a mixed population of rods and cones. This was confirmed by probing companion paraffin-embedded ocular tissues sections with 1D4 anti-opsin monoclonal antibody (specific for the C-terminal epitope of rod opsin^37,38^) and fluor-tagged peanut agglutinin (PNA; binds selectively to “cone matrix sheaths”, the extracellular matrix that surrounds cone OS^39,40^) (see Supplemental Material, Fig. S1). The apical cytoplasm of the RPE also is heavily congested with numerous phagosomes. Feeding AY9944-treated rats a high-CHOL diet (Fig. 1C) clearly afforded substantial protection of the retina from degeneration: the ONL appears much more normal than in Fig. 1B, with 8–10 vertical rows of photoreceptor nuclei— although the packing density appears to be less tight than the control (*cf*. Fig. 1A), suggesting some photoreceptor cell loss, and the rod IS and OS layers appear more robust (although it was difficult to obtain optimal alignment). However, the RPE apical cytoplasm is still markedly congested with phagosomes. Most notably, the image of the retina from an AY9944-treated rat fed a diet with combined CHOL plus antioxidant supplementation (AY3 group; Fig. 1D) was remarkably similar to that obtained from the untreated control rat (*cf*. Fig. 1A), having a full complement of ONL nuclei (11–12 vertical rows), and healthy-looking IS and OS layers. Despite these improvements, however, the RPE cytoplasm still exhibits a substantial accumulation of phagosomes.

These qualitative observations were further augmented by quantitative morphometric analysis, focusing on ONL thickness as a general measure of photoreceptor viability. Figure 1 (*lower* panels, E–F) compares ONL thickness measurements taken in a region between 0.5–2.0 mm from the optic nerve head, along the vertical meridian, in the superior retina (Fig. 1E), inferior retina (Fig. 1F), and the average ONL thickness in both hemispheres (Fig. 1G). Compared with retinas from untreated control eyes (*black* bars), the ONL of retinas from AY1 group rats (*red* bars) was only ∼40–45% as thick (*p* < 0.001), indicating massive death and loss of photoreceptor cells, consistent with the qualitative results shown in Fig. 1B and with previously published results^16,18^. The extent of photoreceptor loss under the conditions employed was comparable in both the superior and inferior hemispheres of the eye (*i*.*e*., the retinal degeneration was symmetrical). Feeding AY9944-treated rats a high-CHOL diet (AY2 group; *blue* bars) yielded a substantial (∼30–35%) and statistically significant (*p* < 0.005) sparing of ONL thickness loss; however, the rescue effect was not complete, *i*.*e*., ONL thickness in retinas from AY2 group rats was still ∼10–15% less (*p* < 0.005) than that of untreated control retinas. However, notably, ONL thickness of retinas from AY9944-treated rats fed a high-CHOL diet supplemented with antioxidants (AY3 group; *green* bars) was not significantly different from that of untreated control retinas (compare green *vs*. black bars, Fig. 1E–G), demonstrating a remarkable and complete sparing of photoreceptor cell loss. Thus, the diet supplemented with CHOL plus antioxidants (AY3) was therapeutically superior (with statistical significance) to the diet supplemented with CHOL alone (AY2), with respect to preservation of retinal morphology.

**Figure 2:**
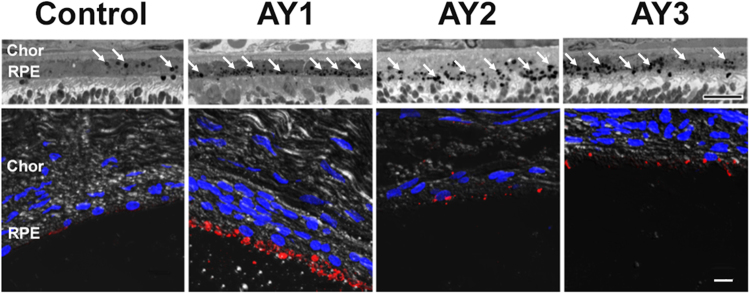
Persistence of phagosomes in RPE of AY9944-treated rats. *Upper panels*: Higher-magnification light microscopy images of RPE (resin embedment; 100X oil objective and 3X digital zoom) at PN 80 days, under same dietary conditions as in Fig. 1. White arrows denote presumed phagosomes in RPE. *Lower panels*: Correlative immunohistochemistry (paraffin embedment; confocal fluorescence microscopy), using anti-opsin (1D4 epitope) and fluor-conjugated secondary IgG (*red*) to label phagocytized photoreceptor outer segment tips in RPE. DAPI counterstain (*blue*). Note the relative paucity of 1D4-positive material in the control RPE, compared to the marked amount of such material in the RPE in AY9944-treated rats. Scale bars, 10 μm. *Abbreviations*: Chor, choroid; RPE, retinal pigment epithelium.

To confirm the identity of the presumed phagosomes in the RPE, we examined the plastic resin-embedded tissue section images at higher magnification (*upper* panels, Fig. 2) by light microscopy, and also examined companion paraffin-embedded tissue sections that had been probed with 1D4 anti-opsin monoclonal antibody, with detection using a fluor-tagged anti-mouse secondary antibody and DAPI counterstain (*lower* panels, Fig. 2), using confocal fluorescence microscopy. For the latter, we chose regions of retina in which the neural retina had separated, capriciously, from the RPE, thereby affording the ability to image the RPE without interference from adjacent photoreceptor OS. Light microscopy confirmed the presence of numerous dark-staining, round inclusions, consistent in morphology and location with phagosomes; clearly, the RPE in eyes from AY9944-treated rats (AY1-AY3) contained considerably more such cytoplasmic inclusions than the RPE in eyes from untreated control rats. In agreement with these findings, 1D4 immunostaining was relatively sparse in the RPE of eyes from control rats, but was intense and densely distributed broadly in the apical cytoplasm of the RPE in eyes from AY1 group rats. Qualitatively, in these specimens, the amount and distribution of 1D4-positive inclusions in the RPE appeared to be less in eyes from AY2 and AY3 group rats, relative to AY1 rat eyes, but was still prominent and also was greater than in the untreated control eyes.

### Combined CHOL-antioxidant dietary supplementation preserves retinal electrophysiological function

**Figure 3:**
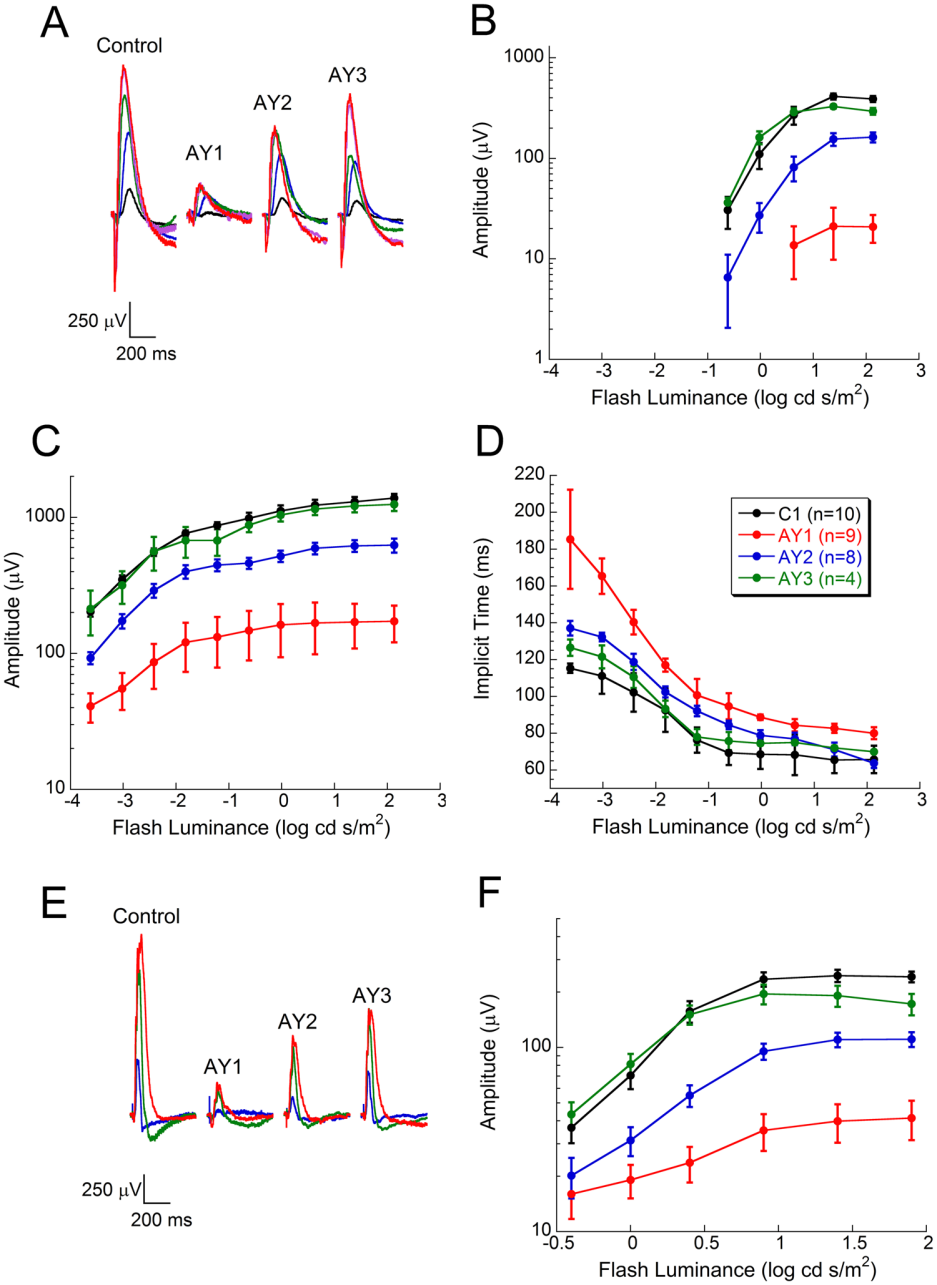
Electroretinographic analysis of control *vs*. AY9944-treated rats as a function of diet. (**A**) Representative dark-adapted ERG waveforms from a member of each experimental group. Flash strength is color-coded: (*black*: −3.6 log cd s/m^2^; *blue*: −2.4 log cd s/m^2^; *green*: −1.2 log cd s/m^2^; *red*: 0.0 log cd s/m^2^; *red*: 1.4 log cd s/m^2^). Summary plots are shown for (**B**) the amplitude of the ERG a-wave and (**C**) b-wave, and (**D**) the implicit time of the ERG b-wave. (**E**) Representative light-adapted cone ERG waveforms from a member of each experimental group. Flash strength is color-coded: (*blue*: 0.0 log cd s/m^2^; *green*: 0.9 log cd s/m^2^; *red*: 1.9 log cd s/m^2^). (**F**) Summary plots for the amplitude of the cone ERG. Data points are mean ± S.E.M. values, for n biological replicates, obtained from rats at age PN 80–82 days at time of ERG measurements: C1 group, n = 10; AY1 group, n = 9; AY2 group, n = 8; AY3 group, n = 4 (see inset, *panel* D, for color key). Note the overlap in data points for ERG responses from C1 and AY3 group animals. [See *Results* for presentation of statistical analysis of data].

Response functions for the major components of the rat ERG, measured at age PN 80–82 days (see *Methods*), are shown in Fig. 3. The dark-adapted (*i*.*e*., scotopic, rod-driven) ERG a-wave, which reflects the light-induced closure of cation channels along the rod outer segment^41,42^, was significantly (*p* ≪ 0.0001) reduced in AY1 group rats (AY9944-treated, fed a CHOL-free diet), relative to age/sex-matched control rats on the same diet (C1 group) (Fig. 3B; *cf*. black *vs*. red data sets), consistent with the AY9944-dependent loss of photoreceptors associated with this model (see above, and^20,21^). Dietary supplementation with a high-CHOL diet (Fig. 3B, AY2 group, blue data set) markedly improved scotopic a-wave amplitudes, consistent with the degree of preservation of photoreceptor cells observed histologically, compared to the AY1 group, but the amplitudes were significantly lower than for control (C1 group) rats (*p* < 0.0001). Strikingly, the scotopic a-wave amplitudes obtained from rats fed a diet supplemented with CHOL plus antioxidants (Fig. 3B, AY3 group, *green* data set) were comparable to those of control rats, showing no statistically significant differences (*p* = 0.12), consistent with the marked preservation of photoreceptors observed anatomically (*cf*. Figs 1 and 2). A similar pattern of changes was noted in the scotopic b-wave amplitude measurements (Fig. 3C), where AY1 group ERG amplitudes were significantly reduced in comparison to control (*p* < 0.0001), AY2 group ERG amplitudes were substantially improved as compared to AY1 animals but remained significantly reduced in comparison to control (*p* < 0.0001), and AY3 group ERG amplitudes were not statistically different from control levels (*p* = 0.26). These trends were consistent with the ONL thickness measurements and overall histological appearance of retinas in each of the experimental groups, as shown above (*cf*. Figs 1 and 2). Prior studies demonstrated that the timing of the scotopic ERG b-wave peak was slowed in the AY9944 model^20^. Figure 3D plots the impact of dietary supplementation on the scotopic b-wave implicit time. The peak times of AY1 rats were significantly slower, compared to controls (*p* < 0.0001). Implicit times of AY2 rats were significantly improved (shortened) in comparison to AY1 rats, but remained significantly slower than in controls (*p* < 0.0001). Implicit times of AY3 rats also were improved in comparison to AY2 animals, but remained significantly slower than those of control rats (p < 0.0001). To examine the impact of AY9944 and dietary treatments on the function of the cone-driven visual signaling pathway, we also recorded ERGs with presentation of light stimuli that were superimposed upon a rod-desensitizing (cone-isolating) adapting field (Fig. 3E,F). The pattern of results described immediately above for the dark-adapted (rod-driven) ERG pertained here as well. In comparison to untreated controls, cone b-wave ERG amplitudes were significantly reduced in AY1 group animals (*p *≪ 0.0001), partially normalized, but still significantly reduced (*p *≪ 0.0001), in AY2 group animals, and not significantly different in AY3 group animals (*p* = 0.15).

### Combined CHOL-antioxidant dietary supplementation significantly reduces levels of 7DHC-derived oxysterols in the neural retina

**Figure 4:**
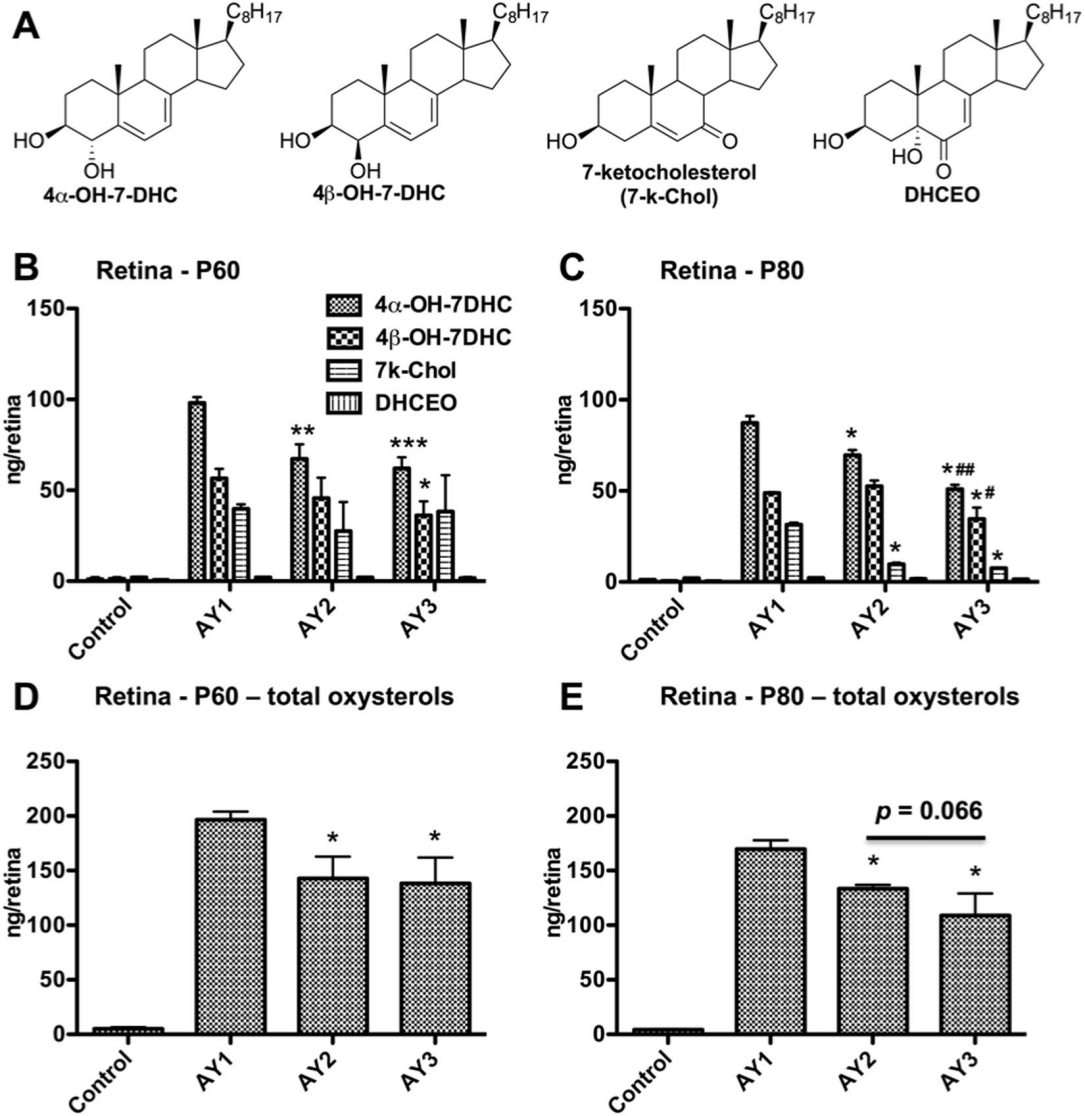
Quantification of selected 7DHC-derived oxysterols in neural retina as a function of dietary regimen and postnatal (PN) age. Oxysterols were quantified by UHPLC-MS/MS as described in Methods; values given in ng/retina. (**A**) Oxysterol structures (see inset, panel B, for key): 4α-hydroxy-cholesta-5,7-dien-3β-ol (4α-OH-7DHC), 4β-hydroxy-cholesta-5,7-dien-3β-ol (4β-OH-7DHC), 7-ketocholesterol (7k-Chol), and 3β,5α-dihydroxycholest-7-en-6-one (DHCEO). Individual oxysterol levels at (**B**) PN 60 days and (**C**) PN 80 days. Total oxysterol levels at (**D**) PN 60 days and (**E**) PN 80 days. For PN 60 days rats, N = 4 for each group. For PN 80 days rats, Control group, N = 4; AY1 group, N = 2; AY2 group, N = 3; AY3 group, N = 3. Significance levels (Student’s *t*-test): *p < 0.05; **p < 0.005, and ***p < 0.001 for comparison of AY1 *vs*. AY2 and AY3 values; ^#^*p* < 0.05 and ^##^*p* < 0.005 for comparison of AY2 *vs*. AY3 values.

Elevated levels of 7DHC-derived oxysterols, including 4α-hydroxy-cholesta-5,7-dien-3β-ol (4α-OH-7DHC), 4β-hydroxy-cholesta-5,7-dien-3β-ol (4β-OH-7DHC), 7-ketocholesterol (7k-Chol), and 3β,5α-dihydroxycholest-7-en-6-one (DHCEO), in the retinas of AY9944-rats have been reported previously^24^. We quantified the levels of these oxysterols (Fig. 4A) in neural retinas from rats at ages PN 60 (Fig. 4B) and PN 80 days (Fig. 4C) as a function of AY9944 treatment and dietary regimen, in comparison with age-matched untreated controls fed a standard rodent chow, by UPLC-MS/MS (see *Methods*). As seen in Fig. 4, the levels of these oxysterols were just barely detectable and quantifiable in neural retinas from untreated control rats, but were markedly elevated in all three treatment groups (AY1, AY2, AY3). At both time points, the dominant oxysterols under all three dietary conditions were the 4α- and 4β-hydroxy derivatives of 7DHC; 7k-Chol was the third most prevalent of the oxysterols examined, while DHCEO levels were just barely detectable. For AY1 group animals, the steady-state levels of these four oxysterols did not appreciably change with age (*cf*. data for PN 60 *vs*. PN 80 days). However, the levels of 4α-OH-7DHC in the retinas of AY2 groups, even more so in AY3 groups, were significantly lower than corresponding AY1 groups at both PN 60 and PN 80 time points. Furthermore, the levels 4β-OH-7DHC were also reduced in retinas from AY3 groups, but not AY2 groups, relative to matching AY1 groups at both time points. Interestingly, the most dramatically altered oxysterol was 7k-Chol: although the levels of this oxysterol were comparable in retinas from AY9944-treated animals across all three dietary groups at PN 60 days, they were dramatically reduced (*ca*. 3- to 5-fold) in both AY2 and AY3 group animals (and to comparable levels) at PN 80 days. A trend of decrease was observed for the level of DHCEO from AY1 to AY2 to AY3 groups, but the apparent changes were not found to be statistically significance (also see Supplementary Material, Table S1). Although DHCEO has been proposed to be a “biomarker” for SLOS in brain of SLOS animal models and in neuronal cells and fibroblasts of SLOS cellular models^17,26^, its level in the retina is very low, resulting in large standard deviations in its measurement in this study, which could account for the lack of statistical significance. Furthermore, we examined the changes to the total oxysterol content as a function of different dietary treatments (Fig. 4D and E). For PN 60 rats, AY2 and AY3 groups exhibited reductions in the total oxysterol levels to a similar extent, relative to those in the AY1 group; however, for PN 80 rats, those in the AY3 group exhibited reductions in total oxysterol levels even further (by *ca*. 36%, relative to AY1 values), as compared to the AY2 group oxysterol levels (*ca*. 21%, relative to AY1) (*p* = 0.066 between AY2 and AY3 group). Taken together, these results are consistent with the initial hypothesis of this study.

In addition, we also measured the steady-state levels of 7DHC and CHOL in neural retinas from AY9944-treated rats, both at PN 60 and PN 80 days, under the three dietary conditions employed in this study. As shown in Table 1, the 7DHC/CHOL mole ratio increased moderately (by *ca*. 22.6%) between PN 60 (4.42 ± 0.74) and PN 80 (5.42 ± 0.15) in retinas from AY9944-treated rats maintained on a standard, CHOL-free rodent diet (AY1 group). These 7DHC/CHOL values are consistent with our previously published findings^20,21^. That ratio was cut approximately in half when animals were fed a high-CHOL diet, at both the PN 60- and PN 80-day time points, with or without dietary antioxidants, largely due to the nearly two-fold increase (ave. 1.94-fold; range: 1.69- to 2.13-fold; *p* < 0.005) in the steady-state levels of CHOL in the retina, as opposed to the relatively modest (ave. 11.7%; range: 8.5–18.2%), but statistically significant (p < 0.05), reductions in 7DHC levels. These observations are also consistent with our prior findings regarding the ability of dietary CHOL to increase the steady-state levels of CHOL in the rat retina in this SLOS animal model^21^, and the ability of blood-borne CHOL to freely cross the blood-retina barrier^43^.

**Table 1:** Effect of a high-CHOL diet, with or without antioxidants, on retina neutral sterol levels in AY9944-treated rats.

Age	Sterol Concentration (µg/retina)^*a*^					
	PN 60 days			PN 80 days		
Diet Group	AY1	AY2	AY3	AY1	AY2	AY3
Chol	4.97 ± 0.85	9.05 ± 0.5**	8.41 ± 0.89**	4.11 ± 0.08	8.68 ± 0.69**	8.78 ± 0.72**
7DHC	21.33 ± 0.65	19.51 ± 0.65*	19.39 ± 1.73	22.26 ± 1.06	19.78 ± 1.49	18.21 ± 1.25
7DHC/Chol	4.42 ± 0.74	2.16 ± 0.11*	2.31 ± 0.13*	5.42 ± 0.15	2.28 ± 0.08*	2.08 ± 0.06^#^

aValues determined by UHPLC-MS/MS as described in *Methods*. N = 3 biological replicates per condition. Statistical significance (Student’s *t*-test), relative to AY1 values: *p < 0.05; **p < 0.005 relative to AY1. ^#^*p* < 0.05 between AY2 and AY3.

### Changes to sterols and oxysterols in liver, serum, and brain of AY9944-rats under different dietary regimens

Similar sterol and oxysterol analyses were carried out on liver, serum, and brain of AY9944-treated rats (see Supplementary Material, Fig. S2). Due to extensive loss of animals over time in the AY1 group, we were not able to obtain replicates for the analysis of liver, serum, and brain regions in this group at the PN 80-day time point. Therefore, only data from PN 60-day time point are presented here. In liver, significant decreases in 7DHC and significant increases in CHOL levels were observed both in AY2 and AY3 group animals relative to those in the AY1 group, resulting in decreases in the 7DHC/CHOL ratio from approximately 31 to 0.45 (Supplementary Material, Table S2). The levels of 7DHC-derived oxysterols also decreased significantly (on average, 75%) in AY2 and AY3 group animals, but no difference was observed between these two groups. Similar changes in the levels of 7DHC and CHOL, and their ratios, were observed in serum as a function of the different diets employed; however, no significant changes in oxysterols were observed between any of the treatment groups and no quantifiable levels of DHCEO were observed in any of the samples analyzed (Supplementary Material, Table S3).

Because CHOL does not cross the blood-brain barrier (see *Discussion*), it is not unexpected to see that the levels of CHOL and 7DHC in different brain regions were not significantly affected in animals fed a CHOL-enriched (AY2 and AY3) diet (Supplementary Material, Fig. S2 and Tables S4 through S7). However, while the oxysterol levels in cortex, brainstem, or cerebellum were not significantly different between the AY groups, their levels in hippocampus were significantly reduced by antioxidant treatment at the PN 60-day time point. It is known that vitamin E does not appreciably cross the brain-brain barrier either (see *Discussion*), which suggests that the decreases in these oxysterols may have been due to the water-soluble antioxidants vitamin C and/or Na-selenite.

## Discussion

The results of the present study both confirm and substantially extend previous observations concerning the AY9944 rat model of SLOS, with regard to the impact of defective cholesterol biosynthesis on the structure and function of the retina and the effectiveness of dietary manipulations that may limit or spare the retina from photoreceptor degeneration under such conditions. It is important to note that the retinal degeneration observed in this animal model is far more severe than the retinal defects observed in human patients afflicted with SLOS^20,44^. This fact makes our findings all the more striking, *i*.*e*., that combined dietary CHOL plus antioxidant supplementation can essentially prevent the massive retinal degeneration that otherwise would occur in this model, and highlights its utility for testing additional therapeutic interventions.

Previously, we demonstrated a progressive (time/age-dependent), irreversible retinal degeneration in the AY9944 rat SLOS model^20,21^, and also showed that the severity of the retinal degeneration was massively exacerbated by exposure of AY9944-treated rats to intense, constant light, an effect which was minimized by pretreatment of the rats with a systemically administered free radical scavenger^45^. The severity of the light-induced retinal degeneration in this rat model of SLOS also correlated with a marked elevation in the levels of lipid hydroperoxides in the retina^46^. Our initial morphological study of the retinas of AY9944-treated *vs*. age-matched control rats suggested that the degeneration and cell death were largely confined to photoreceptor cells. Those findings were confirmed and extended by subsequent TUNEL analysis, which demonstrated that virtually all of the TUNEL-positive cells in the degenerating retina of AY9944-treated rats were localized to the photoreceptor layer^25^. These observations led us to speculate that something present in the retinas of AY9944-treated rats (most likely 7DHC, or a product derived therefrom), but not in untreated control retinas, was extremely sensitive to light and oxygen, promoting the formation of oxidation products that were toxic to retinal cells, particularly photoreceptor cells.

That notion was bolstered by the subsequent discovery that 7DHC is the most reactive lipid molecule toward free radical oxidation known to date^7^, far more so than what one would predict from the number of double bonds in this sterol (*e*.*g*., 7DHC has two double bonds, yet it oxidizes seven times faster than does docosahexaenoic acid (C22:6, n-3), which has six double bonds). Furthermore, it has been demonstrated that oxidation of 7DHC gives rise to more than a dozen unique oxysterols, several of which are highly toxic to cells in culture^8,10,11^. Subsequently, we showed that such 7DHC-derived oxysterols are present in the retina as well as in other tissues in the AY9944 rat SLOS model^9,24^. Such oxysterols are also found in tissues from SLOS patients^29,30^ and from a genetic mouse model of SLOS^26,28^. More recently, we’ve shown that 661 W cells — an SV40 T-antigen transformed cell line derived from mouse cone photoreceptor cells^47,48^, which serves as a convenient *in vitro* surrogate for primary retinal photoreceptor cells — are extremely labile to certain 7DHC-derived oxysterols, far more so than are other retina-derived cells or cell lines (*e*.*g*., rMC-1 retinal Müller glial cells, primary monkey RPE cells)^11^. This latter result is fully consistent with our previously published findings^20,21,25^ and the present work (see Fig. 1, above), which demonstrate that photoreceptors are the primary, if not sole, cell type that undergoes progressive irreversible degeneration and death in the retina in the AY9944 rat SLOS.

In a study conducted a decade ago^21^, we showed that feeding AY9944-treated rats a diet enriched in CHOL (2%, by wt., in the chow) provided a significant improvement in the electrophysiological responses of the retina to light stimuli, particularly with regard to cone-driven responses (the rod-driven responses were not as dramatically improved in that study). However, this only minimally reduced, but did not prevent, the progressive histological damage to the retina. In the present study, we found that feeding a similar high-CHOL diet not only provided more profoundly beneficial effects on both rod and cone function (see Fig. 3, above), but also provided substantial (although incomplete) sparing of photoreceptor cell degeneration and death (see Fig. 1, above). At present, we cannot explain why the high-CHOL diet employed in the current study was so much more therapeutically effective than that used in our prior study^21^.

We have formally proposed that a significant factor in the pathobiological mechanism underlying retinal degeneration in the AY9944 rat model of SLOS (as well as in SLOS itself) is the formation and presence of cytotoxic oxysterols derived from 7DHC^8,10,16,18,34,35^. Clinical studies have shown that CHOL supplementation alone does not provide a reliably efficacious therapeutic intervention for SLOS, and combination of CHOL with statin treatments lead to variable results^49–53^. A very recent report by Wassif *et al*.^53^ which appeared more than a year after the present study commenced, has provided encouraging new evidence for safety and efficacy of simvastatin as a therapeutic intervention for SLOS, with respect to lowering the serum 7DHC/total sterol ratio to some extent and improving irritability behavior. However, in that study, the 7DHC/total sterol ratio was not improved in cerebrospinal fluid samples and no improvement was observed with regard to overall behavior or cognition of patients, based on the Clinician’s Global Impression–Improvement scale. The incomplete rescue of the phenotypes might be due to limited lowering of the 7DHC/total sterol ratio and/or persistence of 7DHC-derived oxysterols. Hence, we reasoned that in order to provide a more substantial and reliable therapeutic outcome, in SLOS patients or in animal models of SLOS such as the AY9944 rat model, one must block or minimize the formation of the oxidation products derived from 7DHC, in addition to exogenously providing CHOL, whose formation is deficient in SLOS and SLOS-like animal models. Hence, in the present study, we tested whether or not a diet enriched with CHOL plus antioxidants (in this case, high levels of vitamins E and C plus Na-selenite, *i*.*e*., the AY3 group) would provide an improvement over a CHOL-rich diet alone (AY2 group) with respect to preservation of retinal structure and function. From the results presented here, the answer to that question is “Yes”. Comparing the histological results (Fig. 1) obtained with the AY2 *vs*. AY3 dietary regimens, the AY3 diet provided substantial and statistically significantly greater preservation of ONL thickness as well as more normal morphological appearance of the photoreceptor IS and OS layers, to a level indistinguishable from that of untreated controls (C1 group). In addition, the robustness of the ERG responses (both scotopic and photopic) was significantly improved (and comparable to control levels) in AY3 group rats compared to those in the AY2 group. Furthermore, the presence of antioxidants in the AY3 group diet resulted in a substantial and statistically significant reduction in the levels of 7DHC-derived oxysterols in the retina, relative to the levels of those compounds observed in retinas from both AY1 and AY2 group rats, yet still substantially greater than in retinas from untreated control rats.

We note that although the levels of CHOL, 7DHC, and 7DHC-derived oxysterols in liver were corrected, to a large extent, by a high-CHOL diet, antioxidant treatment did not further decrease the levels of oxysterols. This is somewhat surprising, since dietary and circulating antioxidants are readily available to the liver without any barrier. However, the levels of oxysterols decreased by approximately 75% in liver in rats fed a high-CHOL diet alone (AY2 group), relative to the AY1 group (CHOL-free diet), much more so than the reduction in oxysterol levels observed in the neural retina, and this reduction appeared to mostly reflect the decreased levels of their precursor, 7DHC (which was decreased by *ca*. 70% in AY2 and AY3 groups, relative to AY1 group animals). This observation suggests that a CHOL-rich diet alone can nearly “normalize” the sterol and oxysterol profiles in the liver of AY9944-treated rats. On the other hand, the sterol and oxysterol levels in the brain regions of cortex, hindbrain, and cerebellum, with the exception of hippocampus, were not significantly affected by the treatments (Fig. S2 and Tables S4 thought S7), which is likely due to the fact that neither CHOL^54^ nor vitamin E^55^ efficiently crosses the blood-brain-barrier. However, it is worth noting that CHOL plus antioxidants (AY3), but not CHOL alone (AY2), led to significant decreases in the levels of oxysterols in hippocampus (Fig. S2 and Table S4). This is an interesting observation, because it suggests that the water-soluble antioxidants vitamin C and selenite may have beneficial effects on the hippocampus, at least in this SLOS animal model. The effect of the water-soluble antioxidants on hippocampus-dependent brain function remains to be investigated. Thus, based on the successful rescue of retinal structure and function and the lowered levels of oxysterols in hippocampus, we suggest that antioxidants that can efficiently cross the blood-brain barrier would provide an effective treatment in lowering the levels of 7DHC-derived oxysterols in the brain, thereby alleviating or dampening their detrimental effects on brain function.

We also note the large differences in oxysterol profiles between different tissue/fluid types. For example, while DHCEO has been established as an effective biomarker for 7DHC peroxidation in human SLOS fibroblasts and brain tissue of SLOS animal models^9,17,26^, its level in retina, liver, and serum is very low. These large differences could arise from different metabolic pathways for DHCEO or its precursor, 7DHC-5α,6α-epoxide. Since many primary 7DHC-derived oxysterols, including 7DHC-5α,6α-epoxide, are highly electrophilic, they are particularly prone to forming adducts with nucleophiles, such as free amino and sulfhydryl groups of amino acids in proteins^56^. Thus, substantial amounts of these electrophilic oxysterols could exist in the form of protein adducts; however, we did not assay for such compounds in the present study. Although we did not observe complete suppression of the formation of detectable 7DHC-derived oxysterols, the essentially complete rescue of retinal structure and function suggests that damage to the retina was prevented by combined CHOL plus antioxidant treatment, in part, possibly by suppressing the formation of adducts between electrophilic oxysterols and nucleophilic biomolecules.

The results presented herein provide proof-of-principle for considering a combined CHOL plus antioxidant regimen as an improved therapeutic intervention for clinical management of SLOS, relative to the current standard of care (CHOL supplementation alone). However, such an extrapolation from this animal model study to humans should be tempered with due caution. First, this combination therapy was applied post-weaning; hence, it did not address the profound embryonic and early neonatal defects that occur in SLOS^1,2,32,33^. Second, unlike the case for the retina, neither CHOL^54^ nor vitamin E^55,57^ crosses the blood-brain barrier, whereas water-soluble molecules such as vitamin C (ascorbic acid) and Se do^57,58^. Indeed, in good agreement with our prior study^21^, we found no statistically significant increase in the steady-state levels of CHOL in most brain regions, except hippocampus, of AY9944-treated rats fed a high-CHOL diet, relative to AY9944-treated rats fed a standard (CHOL-free) rodent diet. Third, there are well-known toxicities associated with high doses of vitamins, including vitamins E and C, particularly when used chronically^59,60^. Moving ahead, however, the outcomes from our study should prompt the design and execution of placebo-controlled clinical trials to develop more effective treatments for SLOS. The results obtained in the present study may be informative in guiding the choice of appropriate combinations of both hydrophobic and hydrophilic antioxidants and micronutrients as adjuncts to CHOL supplementation therapy. In fact, a clinical trial is currently being conducted by Dr. Ellen Elias at the Children’s Hospital Colorado, aiming to test the efficacy of such combination therapy (using fat-soluble vitamins) on improving visual and auditory function of SLOS patients (see ClinicalTrials.gov, Identifier: NCT01773278). Evaluation of the outcome of this clinical trial with suitable biochemical and functional analyses, such as used in the present study, would provide appropriate guidance for a future treatment plan for SLOS patients.

## Methods

### Materials

Unless otherwise stated, biochemical and analytical reagents were used as purchased from Sigma/Aldrich (St. Louis, MO, USA). AY9944 (N-[(2-chlorophenyl)methyl]-1-[4-[[(2-chlorophenyl)methylamino]methyl]-cyclohexyl]methanamine dihydrochloride) was custom synthesized by the Vanderbilt University Chemistry Core (Vanderbilt University, Nashville, TN, USA), and the structure and purity confirmed by ^1^H-NMR and GLC-MS, in comparison with an authentic standard of the compound (a generous gift of Wyeth-Ayerst Laboratories, Radnor, PA, USA). Optima LC/MS grade solvents (methanol, water, formic acid, methylene chloride, and chloroform), sodium chloride, AlexaFluor® 568-conjugated goat anti-mouse IgG, AlexaFluor® 647-conjugated peanut agglutinin (PNA; from *Arachis hypogaea*), and DAPI (4′,6-diamido-2-phenylindole) were purchased from Thermo Fisher Scientific (Grand Island, NY, USA). Mouse monoclonal antibody 1D4, raised against the C-terminal domain of rhodopsin, was purchased from Abcam (Cambridge, MA). Fluoro-Gel™ and other materials and supplies utilized for plastic- and paraffin-embedment microscopy were purchased from Electron Microscopy Science (EMS, Hatfield, PA, USA). Deuterated (d_7_ = [25,26,26,26,27,27,27–^2^H]) sterol standards d_7_-7DHC, d_7_-7-ketocholesterol and d_7_-DHCEO, as well as undeuterated sterols 4α-hydroxy-7DHC and 4β-hydroxy-7DHC, were prepared as reported previously^9,26^. d_7_-CHOL was purchased from Avanti Polar Lipids (Alabaster, AL, USA). Ophthalmic drugs (see *Electrophysiological analysis*, below) were used as obtained from Akorn, Inc (Lake Forest, IL, USA) or Bausch & Lomb Inc. (Rochester, NY, USA).

### Animals

All procedures conformed to the National Research Council’s *Guide for the Care and Use of Laboratory Animals* (https://grants.nih.gov/grants/olaw/Guide-for-the-Care-and-use-of-laboratory-animals.pdf) and the *AVMA Guidelines for the Euthanasia of Animals* (2013 Edition; https://www.avma.org/KB/Policies/Documents/euthanasia.pdf). [See also *Study approval*, below.] Rats were maintained in dim cyclic light (20–40 lux, 12 h light:12 h dark light cycle) at 22–25 °C. The animal model of SLOS employed here was generated by treating rats with AY9944, as previously described^21^. In brief, pregnant adult female Sprague Dawley rats (Harlan Bioproducts for Science, Indianapolis, IN; 6 days post-fertilization) were implanted with an Alzet® osmotic pump (Model 2ML4; Durect Corp., Cupertino, CA) containing a concentrated solution of AY9944 (in 0.01 M PBS, pH 7.4). They were fed a standard laboratory rat diet (Teklad 2016 16% Protein Global Rodent Diet®; Envigo, Madison, WI, USA), which is a plant-based, CHOL-free chow. Pups were injected (*s*.*c*.) three times per week with AY9944 (in 0.01 M PBS, pH 7.4; 30 mg/kg), starting on postnatal day 1 (PN 1) and continuing throughout the experimental time course (up to PN 80–82). In parallel, rats that were not treated at any time with AY9944 were maintained as age-matched controls, on a standard (Teklad 2016 Global 16% Protein Rodent Diet) chow.

### Dietary supplementation with CHOL, with and without antioxidants

Upon weaning (PN 28 days), AY9944-treated rats were randomized into three dietary groups (N = 10–12 each): **AY1**) standard CHOL-free diet (Teklad 2016, per above); **AY2**) high-CHOL diet (Teklad TD.07841; Teklad 2016 rat diet supplemented with 2% (20 g/kg), by wt., CHOL); and **AY3**) CHOL + antioxidant supplemented diet (Teklad TD.150840: Teklad 2016 diet containing 2% CHOL, plus vitamin E (DL-α-tocopheryl acetate (500 IU/g); 1.82 g/kg chow), vitamin C (Stay-C®, ascorbic acid; 1.43 g/kg chow), and sodium selenite pentahydrate (3.4 mg/kg chow)). Animals were maintained on these diets for up to *ca*. 2 months post-weaning.

### Electrophysiological analysis

Electroretinograms (ERGs) were recorded in response to full-field light flashes essentially as described previously^20^. In brief, after overnight dark adaptation, rats were anesthetized with intraperitoneal ketamine (75 mg/kg) and xylazine (5 mg/kg) and their pupils were dilated with eye drops (Mydriacyl® (tropicamide ophthalmic solution, 1% USP); phenylephrine hydrochloride (2.5% ophthalmic solution, USP); cyclopentolate hydrocholoride (1% ophthalmic solution, USP)). Needle electrodes placed in the cheek and tail were used as reference and ground leads, respectively. A thin stainless steel wire active electrode contacted the corneal surface (anesthetized with proparacaine hydrochloride (0.5% ophthalmic solution, USP) eye drops) though a layer of 1% methylcellulose. Light-evoked responses were band-pass filtered (0.3–1000 Hz), differentially amplified, averaged and stored using a UTAS E-2000 (LKC Technologies; Gaithersburg MD, USA) signal averaging system. ERGs were recorded in response to strobe flash stimuli presented in darkness (to record rod-driven signals) and then against a steady rod-desensitizing adapting field (to record cone-driven signals).

### Harvesting and treatment of tissues for analysis

In most instances, at defined time points (see *Results*), rats were euthanatized by carbon dioxide inhalation; alternatively, following ERG recordings, rats were euthanatized by sodium pentobarbital overdose (*ca*. 150 mg/kg, *i*.*p*.). These procedures are in compliance with the *American Veterinarian Medical Association* (*AVMA*) *Guidelines for Euthanasia* (2013 Edn.). For biochemical analyses, neural retinas (free of RPE and choroid) were harvested and flash-frozen in liquid nitrogen in screw-cap polypropylene microfuge tubes (Sarstadt; Newton, MA, USA), then stored at −80 °C in darkness until ready for analysis (see below). Blood was collected via the retrorbital sinus immediately prior to death, and stored in microfuge tubes on ice for *ca*. 45 min; following centrifugation (10 min at 13,000 × *g*; Sorvall Legend Micro-17 tabletop microcentrifuge, Thermo Fisher), the serum was transferred to new microfuge tubes and stored at −80 °C until ready for analysis. Livers and various brain regions (cortex, hippocampus, cerebellum and brainstem) were also harvested and collected for biochemical analyses, as previously mentioned for neural retinas. For histological analysis, eyes were fixed overnight at 4 °C by immersion in a modified Karnovsky’s mixed aldehyde fixative (2% glutaraldehyde, 2% paraformaldehyde, in 0.125 M sodium cacodylate buffer, pH 7.4, containing 0.025% CaCl_2_) after removing the cornea and lens, then transferred to cacodylate buffer and stored at 4 °C prior. Alternatively, for immunohistochemical analysis, freshly enucleated eyes were immersed in PBS-buffered 3.7% formaldehyde (prepared from paraformaldehyde), on ice for 15–30 minutes, and then rinsed in PBS three times. Mixed aldehyde fixed tissue was processed for plastic resin embedment, and formaldehyde-fixed tissue was processed for paraffin embedment and sectioning (see below). Embedment and sectioning eyes for histological and immunohistochemical analysis was performed as described in detail previously^20^, under a fee-for-service agreement with the Research Microscopy and Histology Core, Department of Pathology, Saint Louis University (St. Louis, MO, USA).

### Histological and quantitative morphometric analyses

All eyes were harvested 4–6 h after light onset in the vivarium. In brief, after serial dehydration, eyes were embedded in epoxy resin (Spurrs formulation), histological sections (0.75 μm thickness) were collected onto Superfrost™ Plus glass microscope slides using an ultramicrotome and then stained with Toluidine blue (1%, in 0.1 mM sodium borate), air dried, and coverslipped. Bright-field images were obtained using an Olympus BH-2 photomicroscope (Melville, NY, USA), equipped with an oil-immersion 40X DPlan-Apo objective lens and a digital camera. Phagosome images were acquired using a confocal Leica SPFII microscope, with an oil-immersion 100X objective and 3X digital magnification. Digital images (TIFF files) were stored on a computer and exported to Adobe® Photoshop® for annotation and final assembly for publication. Quantitative morphometric analysis of outer nuclear layer (ONL) thickness was performed essentially as described previously^20^. For each retina, three serial resin-embedded sections were examined, imaged and analyzed. Images were acquired at an interval within 0.5–2.0 mm from the optic nerve head, along the vertical meridian, in the direction of either the inferior or superior hemisphere. For each image, n = 10 ONL thickness measurements (technical replicates) were randomly selected, spanning the length of the image. ONL thickness measurements were calculated by using ImageJ (NIH) software. This provided n = 30 independent replicate ONL measurements per locus; mean ± S.E.M. values were calculated using eyes from n = 3–4 rats (biological replicates) each for control and treatment groups.

### Immunohistochemistry, lectin staining and confocal microscopy

Tissue sections (10-µm thickness) from paraffin-embedded eyes were collected onto Superfrost™ Plus glass microscope slides. Following deparaffinization and rehydration, and then subjected to antigen retrieval as described in detail elsewhere^61^. Briefly, antigen retrieval was performed by incubating deparaffinized sections in sodium citrate buffer (10 mM sodium citrate, 0.05% (v/v) Tween-20, pH 6.0) at 95 °C, for 30 min. For PNA lectin cytochemical staining, sections were incubated with AlexaFluor® 647-conjugated PNA (1:1000 dilution of stock, in TBS supplemented with 0.2% (v/v) Tween-20 (TBST)) for 1 h at room temperature in a humidified chamber. For rhodopsin (carboxy terminus) immunolabeling, sections were first treated with nonimmune (normal) goat serum (5% (v/v) in TBST, containing 0.5% (w/v) BSA, and 0.5% (w/v) fish skin gelatin), in order to block non-specific binding of secondary antibodies (host origin: goat). This was followed by incubation (1 h, at room temperature) with in primary antibody (mouse anti-opsin D4; 1:500, diluted in TBST supplemented with 0.5% (w/v) BSA). After three rinses with TBST, tissue sections were incubated for 45 min at room temperature with AlexaFluor® 568-conjugated goat anti-mouse IgG (1:500 dilution, as above for primary antibody). Slides were rinsed with TBS, counterstained by applying DAPI (working concentration, 1 µg/ml), then rinsed in distilled water, mounted on glass microscope slides using Fluoro-Gel™, coverslipped, and examined with a Leica TCS SPEII DMI4000 scanning laser confocal fluorescence microscope. Images were captured using the 40X oil immersion (RI-1.518) objective under nominal laser intensity (10–20% of maximum intensity), arbitrary gain (800) and offset (−0.2) values, to optimize the signal-to-noise ratio.

### Lipid extraction and UHPLC-MS/MS analyses of sterols and oxysterols

Prior to lipid extraction, internal deuterated standards were added to each sample; the amounts of d_7_-7DHC, d_7_-cholesterol, d_7_-7-ketocholesterol, and d_7_-DHCEO added to each sample, respectively, were as follows: retina–0, 1 µg, 200 ng, and 200 ng; liver–0, 5 µg, 1 µg, and 1 µg; serum–12.5 µg, 12.5 µg, 100 ng, and 100 ng; brain regions–12.5 µg, 12.5 µg, 2 µg, and 2 µg. To extract lipids, approximately 125 mg of liver was homogenized in Folch solution (4 ml CHCl_3_:MeOH (2:1, v/v), containing 1 mM butylated hydroxytoluene (BHT) and triphenylphosphine (PPh3) each, using a blade homogenizer for each sample. NaCl aqueous solution (0.9% (w/v), 1 ml) was added and the resulting mixture was briefly vortexed and then centrifuged for 5 min in a clinical tabletop centrifuge (at ambient temperature). The lower (organic) phase was recovered, transferred to a separate glass tube, and the solvent was removed *in vacuo* using a SpeedVac® (Thermo Fisher Savant). The same procedure was conducted for each of the retina specimen, individually, and each of the four brain regions with approximate tissue weights as follows: cortex, 150 mg; hippocampus, 80 mg; hindbrain, 200 mg; and cerebellum, 150 mg. Finally, the resulting dried tissue extracts were re-dissolved in methylene chloride (1 ml for liver, 0.2 ml for retina, and 1 ml for brain regions) prior to further analysis.

In addition to tissues, rat serum (one 50-µL aliquot per animal) was extracted and the extract was taken to dryness in the same manner as described above. Enzymatic hydrolysis (to convert esterified sterols to free sterols, using cholesterol esterase) was then performed on all serum extracts. The incubation system for hydrolysis consisted of 0.1 M phosphate buffer, pH 7.0, containing 1 unit of cholesterol esterase and 300 µmol of sodium cholate in a total volume of 2 ml, as described previously^62^. The hydrolyzed serum samples were then re-extracted, dried, and redissolved in 500 µl of methylene chloride prior to further analysis. Analysis of sterols and oxysterols were performed by UHPLC-MS/MS using a triple-quadrupole mass spectrometer (API 4000^TM^ or 6500^TM^; AB SCIEX, Ontario, Canada) equipped with atmospheric pressure chemical ionization (APCI). For analysis, an appropriate amount of sample was transferred to an LC vial, dried under a stream of argon, and reconstituted in 90% MeOH with 0.1% formic acid. For sterol analyses, 1:10, 1:2, 1:10, and 1:10 dilution factors from the methylene chloride solutions were used for liver, retina, brain regions, and serum samples, respectively. For oxysterol analysis a 1:1 dilution factor was used for livers and brain regions, and a 2:1 concentration factor was used for retina and serum. Reverse-phase chromatography was performed with the following conditions: C18 column (Kinetex®, 100 mm × 2.1 mm, 1.7 µm particle dia.; Phenomenex, Torrence, CA, USA); flow rate, 0.4 ml/min; elution solvent, 90% MeOH with 0.1% formic acid. MS conditions: spray voltage, 5000 V; curtain gas, 10 psi; ion source gas, 20 psi; collision gas, high; entrance potential, 10 V; collision energy, 25 V; declustering potential, 80.00 V; temperature, 300 °C. For MS analysis, selective reaction monitoring (SRM) was employed to monitor the dehydration process of the ion [M + H]^+^ or [M + H − H_2_O]^+^ as described previously^9,24,26^. 7DHC and cholesterol were quantified relative to d_7_-cholesterol for livers and retinas. For serum and brain regions, 7DHC and cholesterol were quantified relative to the d_7_-7DHC or d_7_-cholesterol internal standards. For all sample types, 4α-hydroxy-7DHC, 4β-hydroxy-7DHC, 7-kChol, and 7-OH-Chol were quantified relative to the d_7_-7-ketocholesterol internal standard. DHCEO was quantified relative to the d_7_-DHCEO internal standard.

### Statistics

For ERG analyses, statistical analysis of luminance-response functions employed a two-way repeated measures analysis of variance (ANOVA). For quantitative morphometric analyses, statistical analysis employed and a one-way ANOVA using PRISM® statistical analysis software (GraphPad Software Inc., La Jolla, CA, USA), also with a *p*-value < 0.05 as the statistical significance threshold. Statistical analysis of sterol and oxysterol data was carried out between different groups using Student’s *t-*test in Microsoft Excel.

### Study approval

The present animal-based studies were reviewed and approved with primary oversight by the Institutional Animal Care and Use Committees (IACUCs) of the VA Western New York Healthcare System and the University at Buffalo (SJF; protocol 466360, AWAN A3354-01) and the Cleveland Clinic Foundation (NSP; protocol 2016-1556, AWAN A3047-01). The authors confirm that all experiments were performed in accordance with relevant guidelines and regulations (see *Animals*, above).

### Data availability

No datasets were generated or analysed during the current study.

## Electronic supplementary material

Supplementary Material
