# Author Correction: Prevention of Retinal Degeneration in a Rat Model of Smith-Lemli-Opitz Syndrome

**DOI:** 10.1038/s41598-018-22647-5

**Published:** 2018-03-06

**Authors:** Steven J. Fliesler, Neal S. Peachey, Josi Herron, Kelly M. Hines, Nadav I. Weinstock, Sriganesh Ramachandra Rao, Libin Xu

**Affiliations:** 1 Research Service, VA Western New York Healthcare System, Buffalo, NY USA; 2 Departments of Ophthalmology and Biochemistry, and Neuroscience Program, Jacobs School of Medicine & Biomedical Sciences, University at Buffalo-The State University of New York (SUNY), Buffalo, NY USA; 3 SUNY Eye Institute, Buffalo, NY USA; 4 Research Service, Louis Stokes Cleveland VA Medical Center, Cleveland, OH USA; 5 Department of Ophthalmic Research, Cole Eye Institute, Cleveland Clinic Foundation, Cleveland, OH USA; 6 Department of Ophthalmology, Cleveland Clinic Lerner College of Medicine of Case Western Reserve University, Cleveland, OH USA; 7 Department of Medicinal Chemistry, School of Pharmacy, University of Washington, Seattle, WA USA; 8 Hunter James Kelly Research Institute, Jacobs School of Medicine & Biomedical Sciences, University at Buffalo- The State University of New York (SUNY), Buffalo, NY USA

Correction to: *Scientific Reports* 10.1038/s41598-018-19592-8, published online 19 January 2018

This Article contains an error in Figure 1 where panels C and D are reversed. The correct Figure 1 appears below as Figure 1.

**Figure 1:**
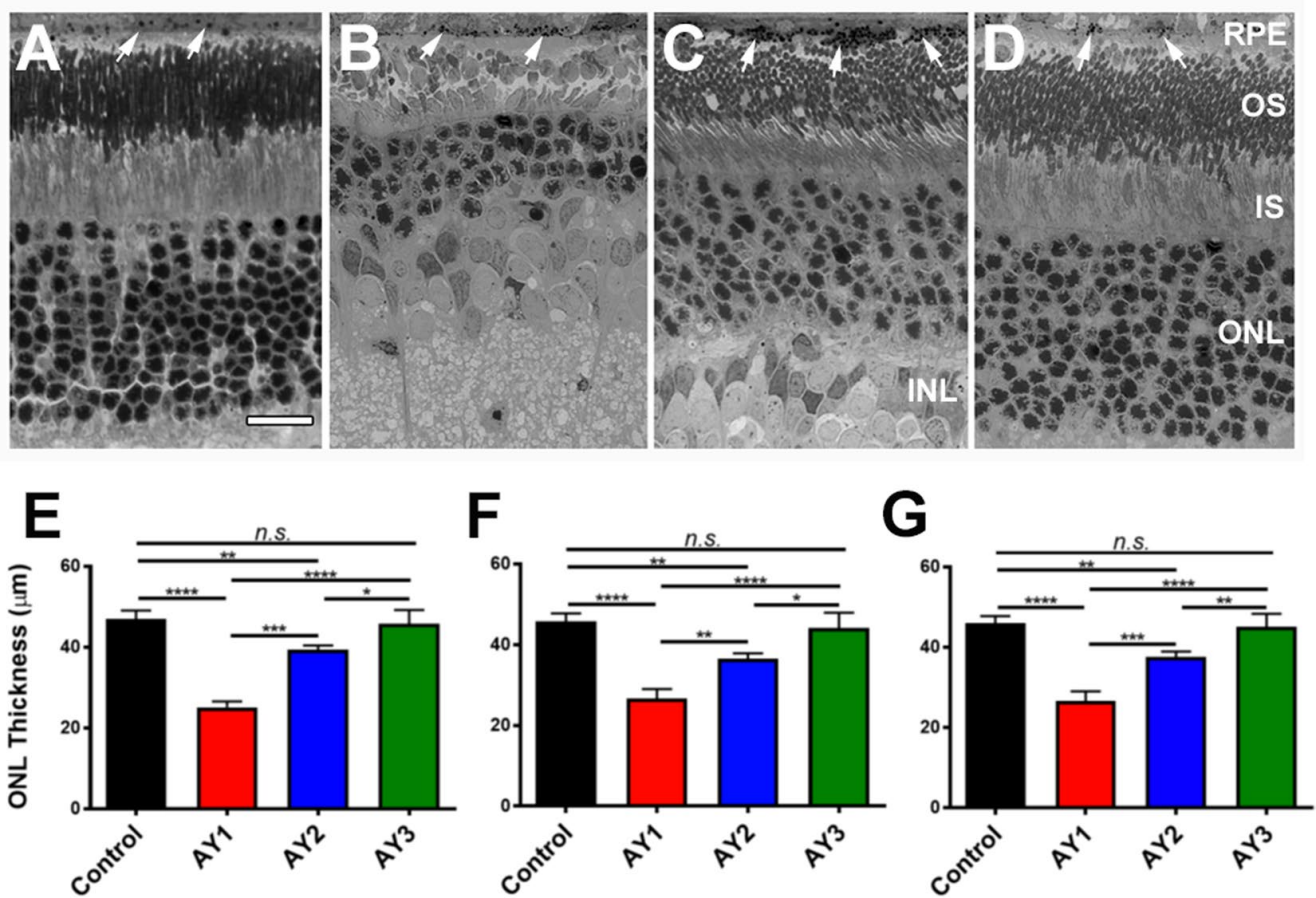
Retinal histology (*upper panels*, *A*–*D*) and quantitative morphometric analysis of ONL thickness (*lower panels*, *E*–*G*) of control *vs*. AY9944-treated rats on various diets. Light microscopy images (resin embedment, Toluidine blue stain; 40X objective) at age PN 80 days, under the following conditions: (**A**) Untreated rat fed a standard rodent diet (C1 group; *black*); (**B**) AY9944-treated rat fed a CHOL-free rodent diet (AY1 group; *red*); (**C**) AY9944-treated rat fed high-CHOL diet (AY2 group; *blue*); and (**D**) AY9944-treated rat fed high-CHOL diet supplemented with antioxidants (AY3 group; *green*). Presumed phagosomes in RPE are denoted by white arrows. *Abbreviations*: RPE, retinal pigment epithelium; OS, outer segment layer; IS, inner segment layer; ONL, outer nuclear layer; INL, inner nuclear layer. Scale bar (*panel* A, for all panels), 20 μm. ONL thickness measurements from (**E**) superior hemisphere, (**F**) inferior hemisphere, and (**G**) combined mean values (both hemispheres, ±S.E.M.; n = 3–4 biological replicates, n = 10 technical replicates, each condition), along the vertical meridian. Statistical significance (one-way ANOVA): *p < 0.05, **p < 0.01, ***p < 0.005, ****p < 0.001; n.s., not significant.

Additionally, this Article contains errors in the Methods section under subheading ‘Lipid extraction and UHPLC-MS/MS analyses of sterols and oxysterols’.

“Analysis of sterols and oxysterols were performed by UHPLC-MS/MS using a triple-quadrupole mass spectrometer (API 4000^TM^ or 6500^TM^; AB SCIEX, Ontario, Canada) equipped with atmospheric pressure chemical ionization (APCI).”

should read:

“Analysis of sterols and oxysterols were performed by UHPLC-MS/MS using a triple-quadrupole mass spectrometer (API 4000™ or 6500™ (for retina and serum oxysterols only); AB SCIEX, Ontario, Canada) equipped with atmospheric pressure chemical ionization (APCI).”

“MS conditions: spray voltage, 5000 V; curtain gas, 10 psi; ion source gas, 20 psi; collision gas, high; entrance potential, 10 V; collision energy, 25 V; declustering potential, 80.00 V; temperature, 300 °C.”

should read:

“MS conditions: declustering potential, 80 V; entrance potential, 10 V; collision energy, 25 V; collision cell exit potential, 20.0 V. APCI parameters: nebulizer current, 3 mA; temperature, 300 °C; curtain gas, 10 psi for 4000™ and 20 psi for 6500™; ion source gas, 20 psi for 4000™and 55 psi for 6500™.”

Finally, the Acknowledgements section in this Article is incomplete.

“This work was supported, in part: by U.S.P.H.S. (NIH) grants R01 EY007361 (SJF), R00 HD073270 (LX), and R01 HD092659 (LX); by Clinical and Translational Science Award UL1 TR001412 to the University at Buffalo-The State University of New York from NCATS/NIH (SJF); by a Foundation Fighting Blindness Center Grant (an unrestricted award to the Department of Ophthalmology, Cleveland Clinic Lerner College of Medicine of Case Western Reserve University) (NSP); by a Research to Prevent Blindness Unrestricted Grant to the Department of Ophthalmology, University at Buffalo-The State University of New York from Research to Prevent Blindness (SJF); by startup funds from the Department of Medicinal Chemistry, School of Pharmacy, University of Washington (LX); and by facilities and resources provided by the VA Western New York Healthcare System (SJF) and the Louis Stokes Cleveland VA Medical Center (NSP).”

should read:

“This work was supported, in part: by U.S.P.H.S. (NIH) grants R01 EY007361 (SJF), R00 HD073270 (LX), and R01 HD092659 (LX); by Clinical and Translational Science Award UL1 TR001412 to the University at Buffalo-The State University of New York from NCATS/NIH (SJF); by a Foundation Fighting Blindness Center Grant (an unrestricted award) to the Department of Ophthalmology, Cleveland Clinic Lerner College of Medicine of Case Western Reserve University (NSP); by Research to Prevent Blindness Unrestricted Grants to the Departments of Ophthalmology, University at Buffalo-The State University of New York and the Cleveland Clinic Lerner College of Medicine of Case Western Reserve University from Research to Prevent Blindness (SJF, NSP); by startup funds from the Department of Medicinal Chemistry, School of Pharmacy, University of Washington (LX); and by facilities and resources provided by the VA Western New York Healthcare System (SJF) and the Louis Stokes Cleveland VA Medical Center (NSP).”

