# Supplementary material for: Prevention of Retinal Degeneration in a Rat Model of Smith-Lemli-Opitz Syndrome
Source: Sci Rep. 2018 Jan 19;8:1286. doi: 10.1038/s41598-018-19592-8 (PMC5775248; doi:10.1038/s41598-018-19592-8)
Supplement: Supplementary file 1 — Supplementary Material [file 41598_2018_19592_MOESM1_ESM.pdf]

Supplementary Material for

Prevention of Retinal Degeneration in a Rat Model of Smith-Lemli-Opitz Syndrome

Steven J. Fliesler,[1,2,3§] Neal S. Peachey,[4-6] Josi Heron,[7] Kelly M. Hines,[7] Nadav I. Weinstock,[8] Sriganesh Ramachandra Rao,[1,2,3] and Libin Xu[7§]

[1]Research Service, VA Western New York Healthcare System, Buffalo, NY (USA); [2]Departments of Ophthalmology and Biochemistry, Jacobs School of Medicine & Biomedical Sciences, University at Buffalo- The State University of New York (SUNY), Buffalo, NY (USA); [3]SUNY Eye Institute, Buffalo, NY (USA); [4]Research Service, Louis Stokes Cleveland VA Medical Center, Cleveland, OH (USA); [5]Ophthalmic Research, Cole Eye Institute, Cleveland Clinic Foundation, Cleveland, OH (USA); [6]Ophthalmology, Cleveland Clinic Lerner College of Medicine of Case Western Reserve University, Cleveland, OH (USA); [7]Department of Medicinal Chemistry, University of Washington, Seattle, WA (USA); and [8]Hunter James Kelly Research Institute, Jacobs School of Medicine & Biomedical Sciences, University at Buffalo- The State University of New York (SUNY), Buffalo, NY (USA)

[§] To whom all correspondence should be addressed at: Dr. Steven J. Fliesler, Research Service, Buffalo VAMC, 3495 Bailey Avenue- Mail Stop 151, Buffalo, NY 14215-1129 (USA). Email: fliesler@buffalo.edu or Dr. Libin Xu, Department of Medicinal Chemistry, University of Washington, Health Sciences Bldg., H-172, Box 357610, Seattle, WA 98195-7610. Email: libinxu@uw.edu

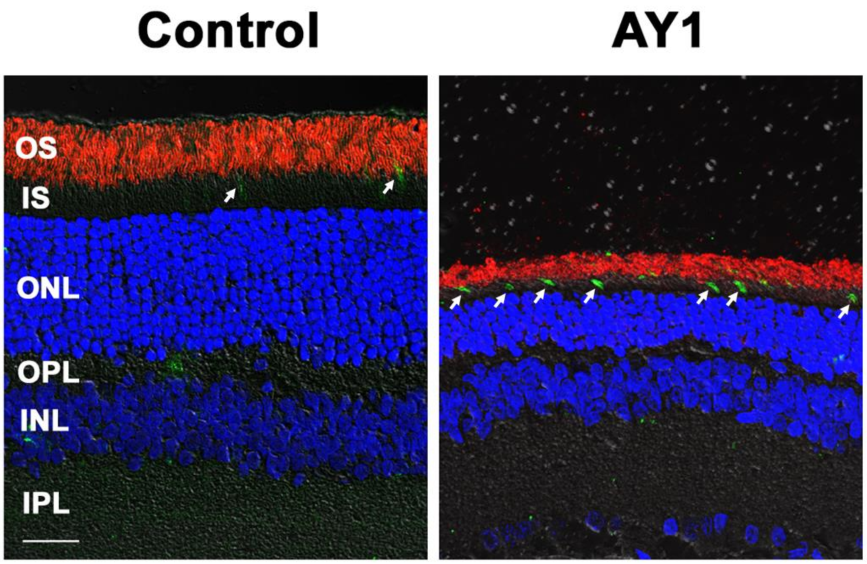

Figure S1. **Confocal fluorescence microscopy images of retinas from control (*left panel*) and AY1 group (*right panel*) rats at PN 80 days**. Rod photoreceptors were labeled using anti-rod opsin 1D4 monoclonal antibody and AlexaFluor® 568-conjugated goat anti-mouse IgG (*red*). Cone photoreceptors were detected using AlexaFluor® 647-conjugated PNA (*green*), which binds to the extracellular matrix that envelopes cone outer segments (*arrows*). *Abbreviations*: OS, outer segment layer; IS, inner segment layer; ONL, outer nuclear layer; OPL, outer plexiform layer; INL, inner nuclear layer; IPL, inner plexiform layer. Scale bar (for both panels), 20 µm.

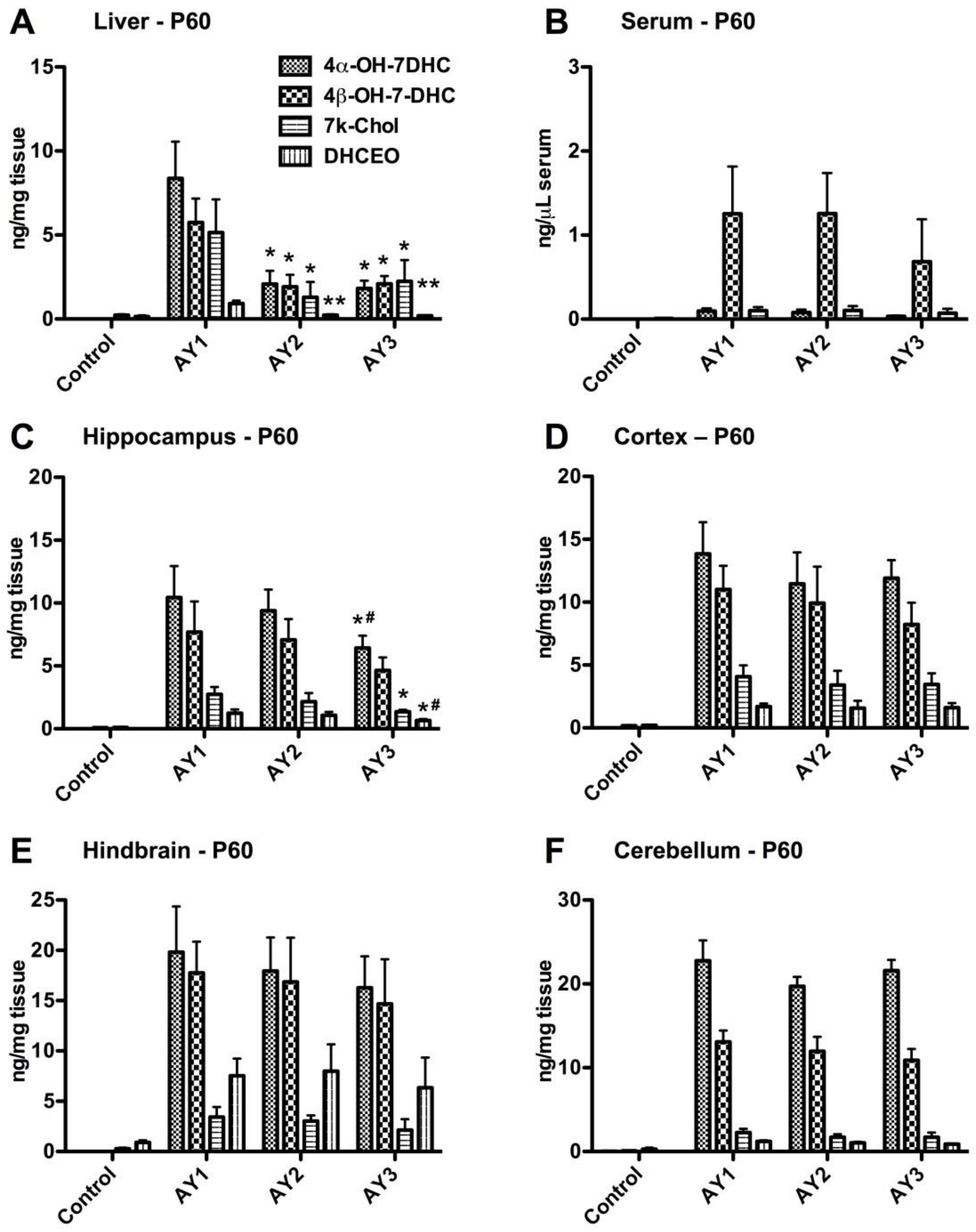

Figure S2. **Quantification of selected 7DHC-derived oxysterols in rat tissues as a function of dietary regimen at PN 60 days.** (**A**) Liver, (**B**) serum, and (**C-F**) brain regions (hippocampus, cortex, brainstem, and cerebellum. See Fig. 4 for legend describing oxysterol structures. N = 4 for each group. *, $p < 0.05$; **, $p < 0.005$, and ***, $p < 0.001$ for comparison of AY1 *vs*. AY2 and AY3 group values; $^{\#}$, $p < 0.05$ and $^{\#\#}$, $p < 0.005$ for comparison of AY2 *vs*. AY3 group values.

Note: For all statistics in the following Tables (S1-S7): *, **, ***, p < 0.05, 0.005, and 0.0005, respectively, relative to AY1. #, ##, p < 0.05 and 0.005, respectively, between AY3 and AY2

Table S1. Sterols and oxysterols in retina samples of AY9944-treated rats at different time points under different diet.

Retina	Postnatal Day 30		Postnatal Day 60				Postnatal Day 80			
	Control	AY1	Control	AY1	AY2	AY3	Control	AY1 (n=2)	AY2	AY3
7-DHC (ug/retina)	0.06 ± 0.06	21.37 ± 2.04	0.03 ± 0.04	21.33 ± 0.65	19.51 ± 0.65 *	19.39 ± 1.73	0.04 ± 0.01	22.26 ± 1.06	19.78 ± 1.49	18.21 ± 1.25
8-DHC (ug/retina)	0.09 ± 0.04	1.99 ± 0.3	0.11 ± 0.03	3.53 ± 0.59	2.14 ± 0.31 *	2.16 ± 0.31 *	0.11 ± 0.02	3.91 ± 0.24	2.02 ± 0.05	1.72 ± 0.26
Cholesterol (ug/ retina)	27.23 ± 1.25	8.55 ± 1.69	26.62 ± 1.87	4.97 ± 0.85	9.05 ± 0.5 **	8.41 ± 0.89 **	26.48 ± 0.46	4.11 ± 0.08	8.68 ± 0.69 **	8.78 ± 0.72 **
4a-OH-7-DHC (ng/ retina)	0.09 ± 0.02	83.97 ± 7	1.02 ± 1.11	98.02 ± 3.33	67.35 ± 7.92 **	62.01 ± 6.15 ***	1.13 ± 0.36	87.31 ± 3.74	69.57 ± 2.9	50.91 ± 2.44 *, ##
4b-OH-7-DHC (ng/ retina)	1.74 ± 0.76	58.96 ± 10.01	1.28 ± 0.94	56.57 ± 5.25	45.71 ± 11.2	36.23 ± 7.69 *	0.57 ± 0.07	48.86 ± 0.51	52.55 ± 3.16	34.55 ± 6.33 #
7-keto-cholesterol (ng/retina)	3.82 ± 1.41	50.13 ± 6.08	2.03 ± 0.36	39.87 ± 2.41	27.62 ± 15.89	38.25 ± 20.07	2.12 ± 0.22	31.5 ± 1.09	9.68 ± 0.85 *	7.62 ± 0.19 *
DHCEO (ng/retina)	1.24 ± 0.38	3.23 ± 0.71	0.66 ± 0.35	2.11 ± 0.21	2.02 ± 0.34	1.64 ± 0.61	0.46 ± 0.18	2.13 ± 0.31	1.73 ± 0.48	1.48 ± 0.09
OH-Chol (ng/ retina)	2.43 ± 0.87	14.6 ± 3.13	1.31 ± 0.19	6.5 ± 1.01	6.79 ± 2.01	7.72 ± 4.54	1 ± 0.1	11.18 ± 5.89	10.25 ± 3.33	4.77 ± 0.7
7-DHC/Cholesterol	0 ± 0	2.57 ± 0.42	0 ± 0	4.42 ± 0.74	2.16 ± 0.11 *	2.31 ± 0.13 *	0 ± 0	5.42 ± 0.15	2.28 ± 0.08 *	2.08 ± 0.06 #

Table S2. Sterols and oxysterols in liver samples of AY9944-treated rats at different time points under different diet.

	Postnatal Day 30		Postnatal Day 60				Postnatal Day 80			
	Control	AY1	Control	AY1	AY2	AY3	Control	AY1 (n=1)	AY2	AY3
7-DHC (ug/mg)	0 ± 0	1.68 ± 0.14	0.01 ± 0.01	2.11 ± 0.17	0.69 ± 0.11 ***	0.72 ± 0.1 ***	0 ± 0	2.18 ± 0	0.78 ± 0.23	0.52 ± 0.16
8-DHC (ug/mg)	0 ± 0	0.08 ± 0.01	0 ± 0	0.26 ± 0.21	0.01 ± 0.01 **	0.01 ± 0 **	0 ± 0	0 ± 0	0.01 ± 0	0.04 ± 0.06
Cholesterol (ug/ mg)	1.6 ± 0.05	0.09 ± 0.02	1.54 ± 0.25	0.07 ± 0.02	1.54 ± 0.17 ***	1.63 ± 0.15 ***	1.53 ± 0.2	0.06 ± 0	1.82 ± 0.06	1.51 ± 0.47
4a-OH-7-DHC (ng/ mg)	0 ± 0	9.07 ± 0.72	0 ± 0	8.36 ± 2.19	2.08 ± 0.78 *	1.82 ± 0.46 *	0 ± 0	7.36 ± 0	1.95 ± 0.34	2.23 ± 0.67
4b-OH-7-DHC (ng/ mg)	0 ± 0	3.96 ± 0.93	0 ± 0	5.74 ± 1.43	1.91 ± 0.72 *	2.1 ± 0.46 *	0 ± 0	3.12 ± 0	2.43 ± 0.75	1.82 ± 0.56
7-keto-cholesterol (ng/mg)	0.17 ± 0.04	4.65 ± 0.78	0.23 ± 0.06	5.14 ± 1.98	1.3 ± 0.91 *	2.24 ± 1.27	0.14 ± 0.03	6.82 ± 0	0.78 ± 0.31	3.35 ± 3.16
DHCEO (ng/mg)	0.02 ± 0.03	0.59 ± 0.12	0.14 ± 0.07	0.92 ± 0.17	0.23 ± 0.05 **	0.24 ± 0.1 **	0.06 ± 0.04	0.65 ± 0	0.28 ± 0.11	0.17 ± 0.13
OH-Chol (ng/mg)	0.31 ± 0.11	8.86 ± 1.02	0.36 ± 0.17	14.3 ± 7.92	2.66 ± 1.22	2.77 ± 0.7	0.36 ± 0.09	6.24 ± 0	3.08 ± 0.35	2.53 ± 0.57
7-DHC/Cholesterol	0 ± 0	18.77 ± 4.38	0.01 ± 0.01	31.4 ± 5.75	0.45 ± 0.05 **	0.44 ± 0.04 **	0 ± 0	37.42 ± 0	0.43 ± 0.13	0.38 ± 0.19

Table S3. Sterols and oxysterols in serum samples of AY9944-treated rats at different time points under different diet.

	Postnatal Day 30		Postnatal Day 60				Postnatal Day 80			
	Control	AY1	Control	AY1	AY2	AY3	Control	AY1 (n=1)	AY2	AY3
7-DHC (ug/uL)	0.01 ± 0	0.24 ± 0.03	0.01 ± 0	0.13 ± 0.04	0.1 ± 0.01	0.08 ± 0.02	0.01 ± 0	0.11 ± 0	0.1 ± 0.01	0.1 ± 0.03
8-DHC (ng/uL)	0 ± 0	9.97 ± 1.8	0 ± 0	6.42 ± 1.04	0.66 ± 1.32 **	0 ± 0 **	0 ± 0	9.72 ± 0	0 ± 0	0 ± 0
Cholesterol (ug/uL)	0.5 ± 0.01	0.01 ± 0	0.32 ± 0.05	0.004 ± 0.001	0.2 ± 0.01 ***	0.19 ± 0.05 **	0.39 ± 0.04	0.003 ± 0	0.21 ± 0.03	0.33 ± 0.08
4a-OH-7-DHC (ng/uL)	0 ± 0	0.3 ± 0.08	0 ± 0	0.1 ± 0.03	0.08 ± 0.03	0.03 ± 0.01 *	0 ± 0	0.1 ± 0	0.05 ± 0.01	0.1 ± 0.07
4b-OH-7-DHC (ng/uL)	0 ± 0	2.5 ± 1.88	0 ± 0	1.25 ± 0.56	1.26 ± 0.48	0.68 ± 0.5	0 ± 0	0.86 ± 0	0.86 ± 0.29	0.83 ± 0.55
7-keto-cholesterol (ng/uL)	0.01 ± 0	0.29 ± 0.05	0.01 ± 0	0.1 ± 0.04	0.1 ± 0.05	0.07 ± 0.05	0.01 ± 0	0.16 ± 0	0.05 ± 0.01	0.32 ± 0.31
DHCEO (ng/uL)	0 ± 0	0 ± 0	0 ± 0	0 ± 0	0 ± 0	0 ± 0	0 ± 0	0 ± 0	0 ± 0	0 ± 0
OH-Chol (ng/uL)	0.04 ± 0.03	0.14 ± 0.03	0.02 ± 0.01	0.04 ± 0.02	0.07 ± 0.03	0.07 ± 0.03	0.01 ± 0	0.04 ± 0	0.09 ± 0.02	0.14 ± 0.06
7-DHC/Cholesterol	0.02 ± 0	23.63 ± 2.03	0.03 ± 0	30.7 ± 4.57	0.53 ± 0.06 **	0.46 ± 0.05 **	0.03 ± 0	37.49 ± 0	0.45 ± 0.06	0.31 ± 0.04 #

Table S4. Sterols and oxysterols in brain hippocampus samples of AY9944-treated rats at different time points under different diet.

	Postnatal Day 30		Postnatal Day 60				Postnatal Day 80			
	Control	AY1	Control	AY1	AY2	AY3	Control	AY1 (n=1)	AY2	AY3
7-DHC (ug/mg)	0.01 ± 0	10.37 ± 3.54	0.02 ± 0.01	8.54 ± 2.17	7.17 ± 0.8	7.86 ± 0.84	0.02 ± 0.01	7.75 ± 0	8.11 ± 1.37	7.48 ± 1.42
8-DHC (ug/mg)	0.02 ± 0	0.28 ± 0.31	0.01 ± 0	0.19 ± 0.03	0.15 ± 0.05	0.17 ± 0.01	0.01 ± 0	0.22 ± 0	0.19 ± 0.01	0.23 ± 0.04
Cholesterol (ug/mg)	10.9 ± 2.1	1.17 ± 0.39	11.79 ± 2.39	1.51 ± 0.19	1.67 ± 0.28	1.36 ± 0.15	10.6 ± 1.21	1.81 ± 0	2.05 ± 0.5	1.86 ± 0.92
4a-OH-7-DHC (ng/mg)	0 ± 0	3.5 ± 0.6	0 ± 0	10.45 ± 2.5	9.4 ± 1.68	6.42 ± 0.99 *,#	0 ± 0.01	6.11 ± 0	7.07 ± 1.27	7.9 ± 2.98
4b-OH-7-DHC (ng/mg)	0.08 ± 0.03	3.46 ± 0.46	0.08 ± 0.02	7.69 ± 2.44	7.07 ± 1.66	4.63 ± 1.04	0.05 ± 0.01	4.09 ± 0	4.54 ± 0.87	4.83 ± 1.66
7-keto-cholesterol (ng/mg)	0.11 ± 0.04	1.05 ± 0.25	0.12 ± 0.04	2.74 ± 0.6	2.16 ± 0.68	1.37 ± 0.15 *	0.1 ± 0.01	1.85 ± 0	0.83 ± 0.21	1.33 ± 0.94
DHCEO (ng/mg)	0 ± 0	0.65 ± 0.09	0 ± 0	1.24 ± 0.3	1.08 ± 0.25	0.66 ± 0.11 *,#	0 ± 0	0.76 ± 0	0.71 ± 0.13	0.67 ± 0.27
OH-Chol (ng/mg)	0.13 ± 0.03	0.5 ± 0.07	0.19 ± 0.07	1.5 ± 0.39	1.33 ± 0.24	0.84 ± 0.15 *,#	0.18 ± 0.02	1.31 ± 0	1.21 ± 0.24	1.13 ± 0.53
7-DHC/Cholesterol	0 ± 0	8.99 ± 1.33	0 ± 0	5.73 ± 1.61	4.44 ± 1.16	5.78 ± 0.47	0 ± 0	4.28 ± 0	4.04 ± 0.62	4.53 ± 1.71

Table S5. Sterols and oxysterols in brain cortex samples of AY9944-treated rats at different time points under different diet.

	Postnatal Day 30		Postnatal Day 60				Postnatal Day 80			
	Control	AY1	Control	AY1	AY2	AY3	Control	AY1 (n=1)	AY2	AY3
7-DHC (ug/mg)	0.02 ± 0	6.47 ± 1.19	0.03 ± 0.01	8.66 ± 1.14	8.87 ± 0.82	7.06 ± 0.89 #	0.04 ± 0.02	6.3 ± 0	6.44 ± 0.89	7.04 ± 0.85
8-DHC (ug/mg)	0.02 ± 0	0.99 ± 1.78	0.01 ± 0	0.18 ± 0.03	0.16 ± 0.04	0.14 ± 0.05	0.01 ± 0	0.2 ± 0	0.14 ± 0.02	0.15 ± 0.02
Cholesterol (ug/mg)	8.54 ± 1.55	1.09 ± 0.22	11.44 ± 1.1	1.44 ± 0.05	2.18 ± 0.58	1.77 ± 0.3	9.28 ± 1.33	2.31 ± 0	2.66 ± 0.27	2.26 ± 0.5
4a-OH-7-DHC (ng/mg)	0 ± 0	10.59 ± 1.75	0 ± 0	13.85 ± 2.51	11.46 ± 2.5	11.9 ± 1.44	0 ± 0	8.69 ± 0	9.24 ± 1.14	9.13 ± 0.76
4b-OH-7-DHC (ng/mg)	0.28 ± 0.04	10.46 ± 3.79	0.18 ± 0.06	11 ± 1.88	9.92 ± 2.91	8.22 ± 1.74	0.07 ± 0.02	5.51 ± 0	6.25 ± 0.7	6.02 ± 0.75
7-keto-cholesterol (ng/mg)	0.23 ± 0.06	3.39 ± 0.58	0.21 ± 0.02	4.07 ± 0.9	3.4 ± 1.14	3.46 ± 0.88	0.16 ± 0.02	5.29 ± 0	1.79 ± 0.07	3.04 ± 1.86
DHCEO (ng/mg)	0.04 ± 0.07	1.99 ± 0.25	0 ± 0	1.69 ± 0.25	1.58 ± 0.57	1.61 ± 0.36	0 ± 0	2 ± 0	1.5 ± 0.09	1.46 ± 0.13
OH-Chol (ng/mg)	0.41 ± 0.14	2.69 ± 0.17	0.35 ± 0.08	3.38 ± 0.77	3.28 ± 1.13	3.49 ± 0.58	0.31 ± 0.05	5.74 ± 0	4.91 ± 0.66	4.27 ± 0.4
7-DHC/Cholesterol	0 ± 0	6.14 ± 1.69	0 ± 0	6.02 ± 0.77	4.29 ± 1.2	4.09 ± 0.87 *	0 ± 0	2.73 ± 0	2.42 ± 0.17	3.26 ± 0.92

Table S6. Sterols and oxysterols in hindbrain samples of AY9944-treated rats at different time points under different diet.

	Postnatal Day 30		Postnatal Day 60				Postnatal Day 80			
	Control	AY1	Control	AY1	AY2	AY3	Control	AY1 (n=1)	AY2	AY3
7-DHC (ug/mg)	0.02 ± 0.01	1.4 ± 0.27	0.03 ± 0.02	1.21 ± 0.09	1.25 ± 0.14	1.27 ± 0.1	0.03 ± 0	1.49 ± 0	1.93 ± 0.55	1.15 ± 0.06
8-DHC (ug/mg)	0.04 ± 0.01	0.15 ± 0.09	0.06 ± 0.02	0.29 ± 0.04	0.25 ± 0.05	0.22 ± 0.02 *	0.05 ± 0.01	0.34 ± 0	0.26 ± 0.11	0.29 ± 0.02
Cholesterol (ug/mg)	15.96 ± 0.93	1.56 ± 0.39	16.5 ± 5.39	1.53 ± 0.06	1.98 ± 0.5	1.52 ± 0.1	18.45 ± 2.02	1.82 ± 0	1.89 ± 0.07	1.88 ± 0.28
4a-OH-7-DHC (ng/mg)	0.06 ± 0.07	13.5 ± 4.3	0 ± 0	19.82 ± 4.54	17.94 ± 3.35	16.29 ± 3.11	0 ± 0	13 ± 0	15.28 ± 5.12	13.47 ± 1.09
4b-OH-7-DHC (ng/mg)	0 ± 0	14.23 ± 3.97	0 ± 0	17.77 ± 3.09	16.87 ± 4.38	14.69 ± 4.43	0 ± 0	11.01 ± 0	13.68 ± 5.23	10.14 ± 0.73
7-keto-cholesterol (ng/mg)	0.93 ± 0.4	4.6 ± 1.31	0.29 ± 0.1	3.42 ± 1	3.02 ± 0.57	2.13 ± 1.09	0.34 ± 0.07	2.67 ± 0	1.1 ± 0.57	2.47 ± 1.98
DHCEO (ng/mg)	0.04 ± 0.02	3.53 ± 1.13	0.02 ± 0.01	2.12 ± 0.34	2.13 ± 0.81	1.44 ± 0.43	0.01 ± 0.01	1.19 ± 0	1.72 ± 0.8	1.87 ± 0.62
OH-Chol (ng/mg)	0.47 ± 0.15	3.31 ± 1.2	0.92 ± 0.2	7.53 ± 1.72	7.99 ± 2.68	6.35 ± 3.01	0.88 ± 0.29	8.68 ± 0	9.96 ± 5.32	14.54 ± 6.98
7-DHC/Cholesterol	0 ± 0	0.95 ± 0.31	0 ± 0	0.79 ± 0.07	0.66 ± 0.17	0.84 ± 0.1	0 ± 0	0.82 ± 0	1.02 ± 0.3	0.62 ± 0.1

Table S7. Sterols and oxysterols in cerebellum samples of AY9944-treated rats at different time points under different diet.

	Postnatal Day 30		Postnatal Day 60				Postnatal Day 80			
	Control	AY1	Control	AY1	AY2	AY3	Control	AY1 (n=1)	AY2	AY3
7-DHC (ug/mg)	0.02 ± 0.01	0.93 ± 0.09	0.02 ± 0	0.83 ± 0.17	0.86 ± 0.13	0.92 ± 0.2	0.03 ± 0.01	0.75 ± 0	0.91 ± 0.14	0.85 ± 0.17
8-DHC (ng/mg)	15.82 ± 1.26	15.11 ± 3.92	15.11 ± 3.92	15.98 ± 2.26	18.11 ± 3.07	19.93 ± 2.88	14.36 ± 2.7	13.08 ± 0	18.9 ± 6.03	0.17 ± 16.24
Cholesterol (ug/mg)	4.63 ± 0.83	0.84 ± 0.2	5.12 ± 1.16	1.07 ± 0.08	1.2 ± 0.2	1.11 ± 0.09	4.84 ± 0.85	1.15 ± 0	1.32 ± 0.08	1.32 ± 0.2
4a-OH-7-DHC (ng/mg)	0.02 ± 0.01	16.58 ± 2.22	0.03 ± 0.01	22.75 ± 2.44	19.72 ± 1.12	21.6 ± 1.26	0.04 ± 0.02	22.46 ± 0	19.93 ± 2.27	20.69 ± 2.21
4b-OH-7-DHC (ng/mg)	0.09 ± 0.01	12.74 ± 0.83	0.08 ± 0.03	13.09 ± 1.35	11.96 ± 1.72	10.88 ± 1.35	0.05 ± 0.02	12.08 ± 0	9.24 ± 1.21	9.04 ± 1.22
7-keto-cholesterol (ng/mg)	0.24 ± 0.01	3.94 ± 0.37	0.29 ± 0.17	2.26 ± 0.45	1.71 ± 0.36	1.72 ± 0.54	0.21 ± 0.01	2.29 ± 0	0.91 ± 0.29	1.53 ± 0.87
DHCEO (ng/mg)	0.02 ± 0.03	2.28 ± 0.55	0 ± 0	1.22 ± 0.12	1.03 ± 0.14	0.89 ± 0.07 **	0 ± 0	1.02 ± 0	0.86 ± 0.14	0.82 ± 0.13
OH-Chol (ng/mg)	0.28 ± 0.03	3.62 ± 0.59	0.44 ± 0.19	5.14 ± 0.54	4.22 ± 0.39 *	4 ± 0.35 *	0.39 ± 0.02	9.53 ± 0	5.87 ± 1.52	4.96 ± 0.43
7-DHC/Cholesterol	0 ± 0	1.16 ± 0.32	0 ± 0	0.79 ± 0.21	0.73 ± 0.18	0.83 ± 0.19	0.01 ± 0	0.65 ± 0	0.69 ± 0.14	0.65 ± 0.17
